# Effect of iron addition to the electrolyte on alkaline water electrolysis performance

**DOI:** 10.1016/j.isci.2023.108695

**Published:** 2023-12-10

**Authors:** Maximilian Demnitz, Yuran Martins Lamas, Rodrigo Lira Garcia Barros, Anouk de Leeuw den Bouter, John van der Schaaf, Matheus Theodorus de Groot

**Affiliations:** 1 Department of Chemical Engineering and Chemistry, Sustainable Process Engineering Group, Eindhoven University of Technology, P.O. Box 513, Eindhoven 5600 MB, the Netherlands; 2 Eindhoven Institute for Renewable Energy Systems, Eindhoven University of Technology, PO Box 513, Eindhoven 5600 MB, the Netherlands

**Keywords:** Electrochemistry, Applied sciences

## Abstract

Improvement of alkaline water electrolysis is a key enabler for quickly scaling up green hydrogen production. Fe is omnipresent within most industrial alkaline water electrolyzers and its effect on electrolyzer performance needs to be assessed. We conducted three-electrode and flow cell experiments with electrolyte Fe and Ni electrodes. Three-electrode cell experiments show that Fe ([Fe] = 6–357 μM; ICP-OES) promotes HER and OER by lowering both overpotentials by at least 100 mV at high current densities (T = 35°C–91°C). The overpotential of a zero-gap flow cell was decreased by 200 mV when increasing the Fe concentration ([Fe] = 13–549 μM, T = 21°C–75°C). HER benefits from the formation of Fe dendrite layers (SEM/EDX, XPS), which prevent NiH_x_ formation and increase the overall active area. The OER benefits from the formation of mixed Ni/Fe oxyhydroxides leading to better catalytic activity and Tafel slope reduction.

## Highlights

• Reduction of HER overpotential by 100 mV with increasing electrolyte Fe concentration
• Reduction of OER overpotential by 200 mV with increasing electrolyte Fe concentration
• Fe dendrites grow during HER and NiFeO(OH) species form during OER operation
• Flow cell tests showed total overpotential reduction of 200 mV; performance gain of 20

## Graphical abstract

**Figure:**
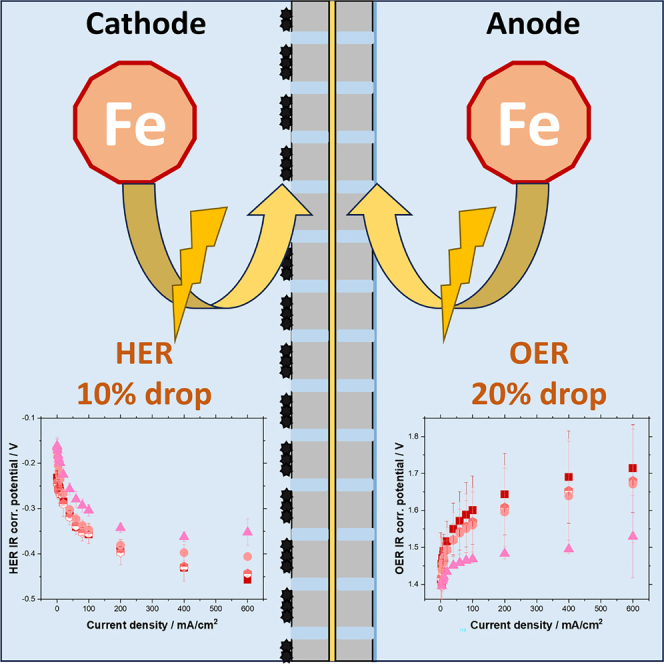

## Introduction

Reducing the usage and global dependence on fossil fuels to end worldwide climate change is one of the key challenges of the current and upcoming decades. Hard to abate industries and transportation, such as steel production, chemical industry, aviation, and international shipping will need hydrogen to de-fossilize. Here, green hydrogen produced from electrolysis using renewable energy will play a key role.^1^ One of the most mature technologies to produce green hydrogen at large scale is alkaline water electrolysis (AWE).^2^^,^^3^^,^^4^ In contrast to proton exchange membrane (PEM) water electrolysis, AWE is not reliant on rare noble metals, such as Pt, Pd, Ru, or Ir.^5^^,^^6^ The most common catalysts employed in AWE are Ni and Fe, either in their pure metal or metal oxide form or in tandem with each other.^7^^,^^8^ In recent years, one of the key aspects surrounding AWE research was to increase its efficiency by lowering the cell potential while simultaneously achieving as high current densities as possible.^7^^,^^9^^,^^10^^,^^11^^,^^12^^,^^13^

One of the main pathways to lower the cell potential, and thus energy consumption, is to employ new types of catalysts and electrodes. In comparison to pure Ni electrodes, Fe addition has shown to be beneficial to the anodic oxygen evolution reaction (OER), but also the cathodic hydrogen evolution reaction (HER).^14^ Fe intermixed with Ni benefits the HER, since it prevents the deactivation of Ni caused by the formation of a Ni-hydride layer on the cathode surface.^15^ For FeNi alloys Flis-Kabulska and Flis observed that an Fe content up to 90% is beneficial to the HER, significantly increasing the cathodic current at similar potentials.^16^ From those results it is not surprising that AWE with 0.5 and especially 14 ppm Fe^3+^ impurities has been shown to benefit the HER.^17^^,^^18^ In particular, at high Fe^3+^ concentrations needle like Fe depositions on the surface can be observed, leading additionally to a higher surface area of the cathode.^18^ Of course Fe poisoning of (noble) metals, especially used on the cathode, might occur as well, reducing the cathodes electrocatalytic activity.^19^^,^^20^

The main focus for AWE research, however, is set on the OER, since the reaction pathway to form O_2_ from OH^−^ is a multi-step process and thus developing active catalysts to improve the overall OER is more challenging.^21^ Studies typically focus on combining non-platinum group metals such as Ni and Fe as catalysts.^22^^,^^23^^,^^24^^,^^25^ Where most literature studies focus on perfecting the ideal Fe to Ni ratio in a catalytic layer, less attention has been paid to what effect dissolved Fe in the electrolyte might have on the performance of AWE. Fe in low concentrations is usually omnipresent during AWE, since it might originate from steel pipes, steel vessels, the electrolyzer itself, KOH impurities, and/or catalytic coatings on the electrodes. The solubility of Fe in concentrated alkaline solutions should be low according to the solubility products (K_sp_(Fe(OH)_2_) = 8 · 10^−16^; K_sp_(Fe(OH)_3_) = 10^−36^).^26^ However, in the past concentrations of 2 ppm Fe have been encountered in the catholyte in industrial size cells for chlor-alkali electrolyzers, while a maximum solubility of up to 60 ppm at 80°C has been reported.^19^ Due to the abundance of hydroxide and the generally oxidic conditions, Fe will form mainly the hardly soluble Fe(OH)_3_. Yet, Fe hydroxides are prone to form colloids, which might enhance their mobility and thus accessibility to the electrodes.^27^^,^^28^ It has been shown, that even in the low ppm range, Fe might be strongly beneficial to the OER.^22^ In a study using pure Ni electrodes with an electrolyte practically devoid of Fe and a 1 ppm Fe electrolyte, Corrigan observed a more than 100 mV higher OER overpotential for the Fe “free” electrolyte.^29^ Similar observations were made by Trotochaud et al., who saw a decrease in activity for Ni(OH)_2_ in Fe free electrolyte compared to electrolyte containing Fe.^30^

Fe containing electrolyte might also prove crucial to the catalyst lifetime. In a recent study Chung et al. observed that catalytic activity for Fe containing Ni- or Co-hydr(oxy)oxide clusters decreased in Fe free electrolyte.^31^ However, with electrolyte containing 0.1 ppm of Fe a self-healing of the catalyst was observed: Fe tends to leach out of the catalyst, however, at the same time Fe from the electrolyte re-adsorbs, maintaining the catalysts activity. Similar self-healing properties of Fe based catalyst within Fe containing electrolyte have also been observed for FeNi-LDH (layered double hydroxides) and FeOOH.^32^^,^^33^

Based on the previous literature studies it seems that Fe in the electrolyte can increase electrocatalytic activity of HER and OER, but also enhances the catalysts self-healing abilities. However, few studies have dived into the topic of an ideal electrolyte Fe concentration. For example, Chung et al. saw that OER activity was not improved in Fe concentrations exceeding 0.1 ppm, but those studies were performed at room temperature and at low current densities (up to 30 mA/cm^2^).^31^ As such, it gives reason to question what electrolyte Fe concentrations might be more optimal at varying current densities and temperatures, especially close to industry conditions?

In this study, we investigate the effect of Fe doping in 30 wt. % KOH in near industrial conditions. We perform three electrode studies to investigate the effect of different concentrations of Fe in the electrolyte on HER and OER on nickel electrodes at different temperatures. From this we can draw conclusions about optimal iron concentration, but also kinetics, and changes of the electrochemical surface area (ECSA). Further, we perform studies in a flow cell with a mixed catholyte and anolyte flow under similar Fe concentrations and temperatures, so we may gain an understanding on how the specific conditions in a flow cell influence the effect of Fe and how electrolyte containing Fe might impact the cell performance in industrial electrolyzers. Throughout the study we monitor the electrolyte Fe concentration using ICP-OES, while utilizing XPS and SEM/EDX to investigate the change in the electrode surface composition.

## Results

### Three electrode studies

#### ICP measurements

The studies in the three-electrode cell were carried out with 30 wt. % KOH with an addition of 0, 25, 50, and 500 μM of Fe in the form of Fe_2_(SO_4_)_3_ hydrate. As soon as the yellow Fe_2_(SO_4_)_3_ hydrate was added to the KOH, it formed hardly soluble dark-red Fe(OH)_3_, which precipitated immediately. At concentrations up to 25 μM it was possible to dissolve the Fe(OH)_3_ by stirring and ultra-sonification treatment. At higher concentrations the Fe(OH)_3_ particles precipitated quickly.

To confirm the amount of Fe present within the electrolyte, we applied ICP (see Table 1). One batch of electrolyte was prepared for each Fe concentration, which was measured before electrolysis. The batch was then split for the HER and OER experiments, and the Fe concentration was measured again at the end of the experiments.

**Table 1:** Fe concentration in the electrolyte pre- and post-electrolysis as determined by ICP-OES

[Fe] sample	/μM	/in ppm (H_2_O)
0 μM Fe pre electrolysis	5.5 ± 1.8	0.10
0 μM Fe HER post	21.9 ± 0.7	0.40
0 μM Fe OER post	14.8 ± 0.6	0.27
25 μM Fe pre electrolysis	19.9 ± 1.0	0.36
25 μM Fe HER post	70.2 ± 11.9	1.26
25 μM Fe OER post	25.3 ± 0.6	0.46
50 μM Fe pre electrolysis	39.8 ± 1.2	0.72
50 μM Fe HER post	22.7 ± 0.1	0.41
50 μM Fe OER post	n.a.	n.a.
500 μM Fe pre electrolysis	357.1 ± 3.4	6.43
500 μM Fe HER post	1092.7 ± 13.1	19.67
500 μM Fe OER post	115.9 ± 21.2	2.09

Pre-electrolysis experiments were conducted before each experimental batch and post-electrolysis experiments after conclusion of the entire experimental batch (experimental batch incl. experiments at all temperatures). n.a., not available. Unfortunately, for 50 μM Fe OER no measurement could be conducted due to an instrumental error and a consequent lack of sample volume.

The results show that trace amounts of 5.5 μM or 0.1 ppm Fe are already present without any added Fe. This Fe originates from the KOH pellets used to prepare the electrolyte, which according to the specifications of the supplier may amount to as much as 10 ppm. These Fe concentrations are already in a concentration range, where significant impact on the OER is expected.^22^^,^^29^ This shows the importance of monitoring the iron concentration in research on OER.

As is obvious from Table 1 the Fe concentration changes during the measurements, either becoming significantly higher or lower. The Fe concentration post electrolysis varies considerably, which can be explained by several factors: Fe electrolyte concentration decreases due to reduction of Fe on the electrode. The concentration may also increase if significant amounts of electrolyte evaporate over extended periods of time (after electrochemical measurement have already been completed; no impact on electrochemical measurements was observed). Fe may also leach from the Ni-201, which can contain up to 2% of Fe. Furthermore, it cannot be completely ruled out that Fe may deposit elsewhere in the setup, increasing the Fe concentration in follow up experiments.

#### Cyclic voltammetry

**Figure 1:**
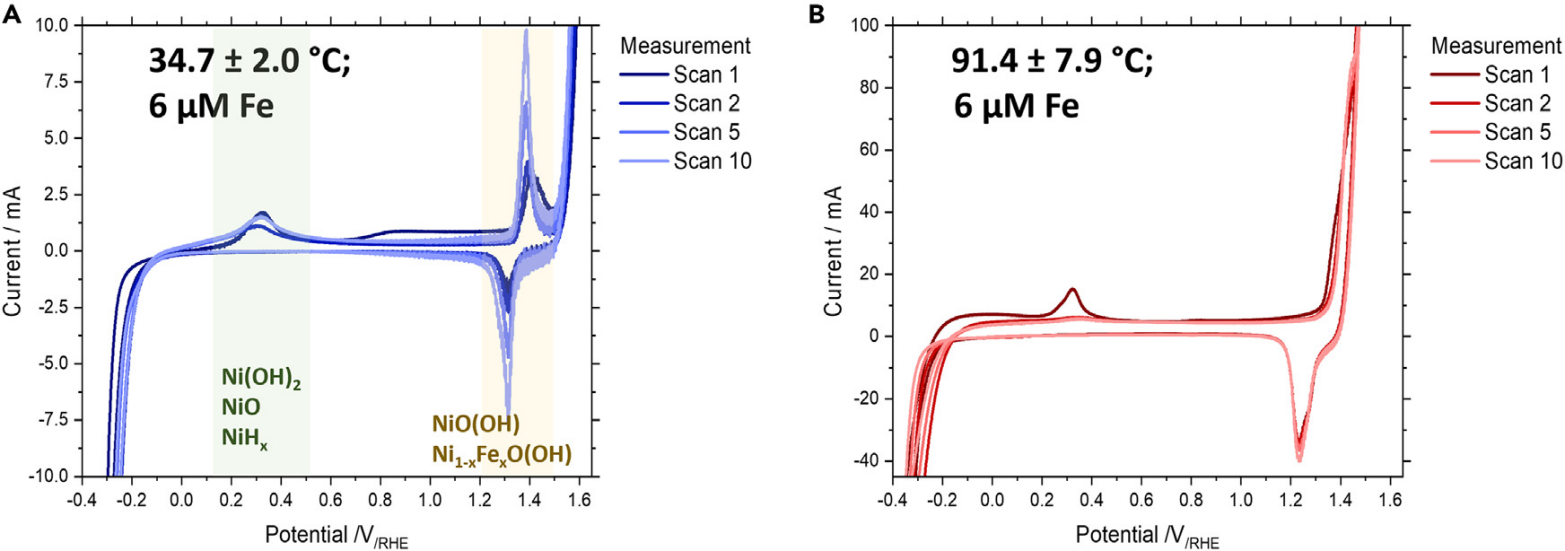
Temperature dependent cyclic voltammograms (A and B) Full cyclic voltammetry scans (V = −0.35 – 1.60 V/_RHE_) for (A) a fresh electrode at 35°C with 6 μM Fe before conditioning and (B) a fresh electrode at 91°C with 6 μM Fe before conditioning showcasing the change of peak locations with increasing temperature.

Full cyclic voltammetry was conducted at each Fe concentration at the start of each experiment at different temperatures using the working electrode either as HER or OER. We could identify two main regions of interest within the cyclic voltammogram: (1) between −0.4 and 0.6 V_/RHE_, where HER, Fe oxidation/reduction, and Ni^0^ oxidation can be identified and (2) between 0.8 and 1.6 V_/RHE_, where Ni^2+^ and Ni^3+^ oxidation/reduction and OER are observed (see Figure 1A). The first CV obtained at 35°C shows a broad peak with a maximum located between 0.27 and 0.33 V_/RHE_. In the literature, it is suggested that this peak corresponds either to the oxidation of incorporated hydrogen in the Ni metal (NiH_x_) or to the formation of a NiO/Ni(OH)_2_ layer.^34^^,^^35^^,^^36^^,^^37^^,^^38^ It has been suggested that first the α-Ni(OH)_2_ forms at 0.3 V_/RHE_, which at higher potentials is electrochemically transformed to the thermodynamically more stable β-Ni(OH)_2_.^39^ In our measurements, we observed that the peak could be seen for multiple cycles at low temperatures, while it quickly disappeared after only a few cycles at higher temperatures. At higher potentials between 1.30 and 1.42 V_/RHE_ the Ni^2+^ species are further oxidized to form NiO(OH) (see Figure 1). Pre-electrolysis CVs at higher Fe concentrations can be found in Figures S4–S6 of the SI.

**Figure 2:**
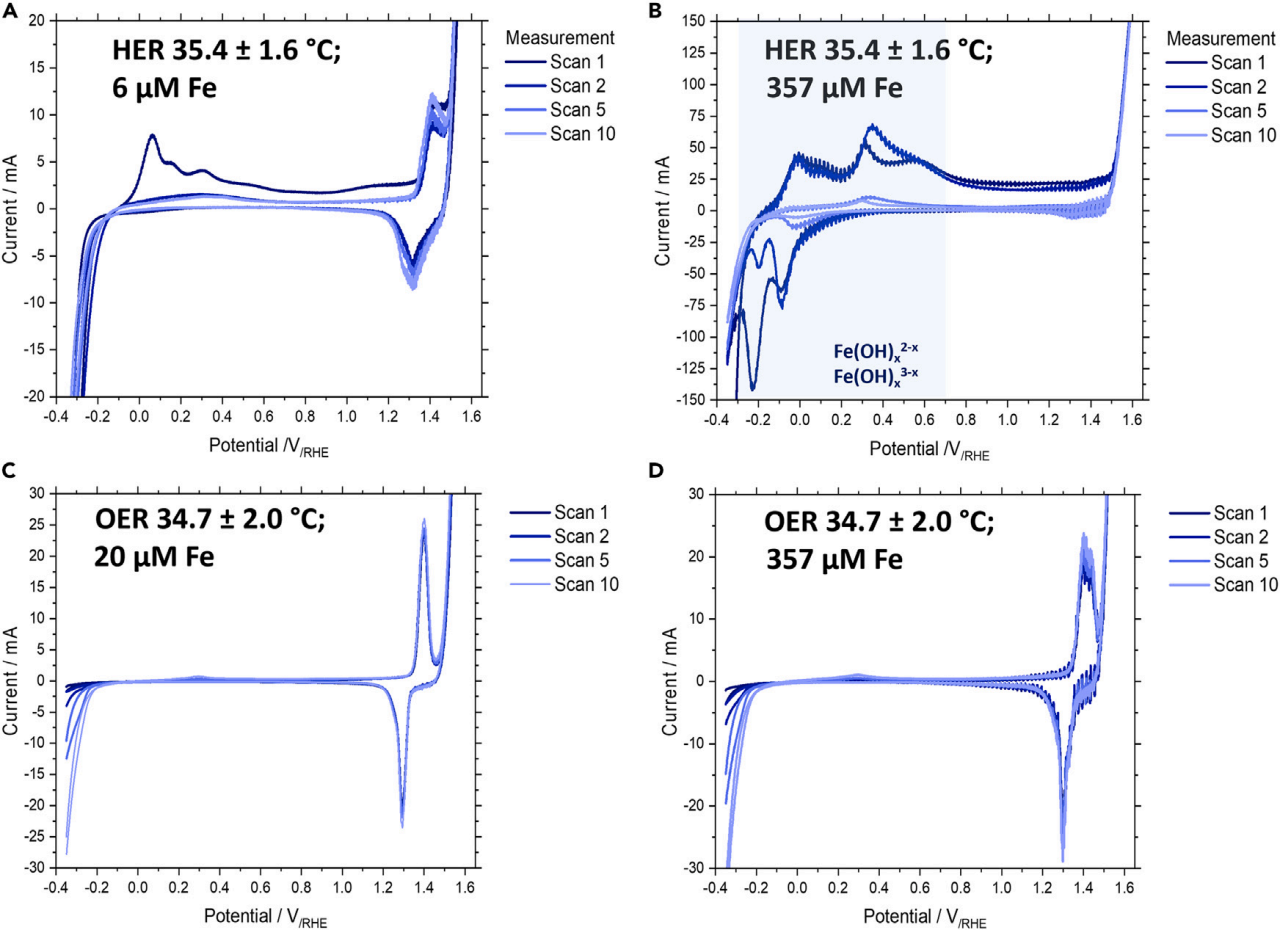
Fe concentration dependent cyclic voltammograms (A–D) Full cyclic voltammetry scans (V = −0.35 – 1.60 V/_RHE_) after HER conditioning at 35°C at (A) 6 μM electrolyte Fe and (B) 357 μM electrolyte Fe showcasing the emergence of Fe hydroxide peaks with increasing Fe concentration. Full cyclic voltammetry scans after OER conditioning at 35°C (C) 20 μM electrolyte Fe and (D) 357 μM electrolyte Fe showcasing the lack of Fe peaks and the slight right shift and increase of intensity of the Ni_1-x_Fe_x_O(OH) peaks.

The working electrodes were then conditioned either for HER or OER for multiple hours and after that CVs were again recorded. For all full CVs, we observe a clear distinction between HER and OER conditioning. For working electrodes conditioned during HER we observe multiple new peaks between −0.2 and +0.6 V_/RHE_ (see Figure 2B). The number of these peaks and their intensity is growing with increasing Fe concentration (see Figures 2A and 2B). As such these new peaks can probably be assigned to the reduction and oxidation of Fe species.^40^ Interestingly, the peaks associated with Fe reactions are decreasing drastically in intensity with each full scan. This effect is likely to be explained by the surface Fe being oxidized and thus forming Fe hydroxide, which can dissolve or disperse into the electrolyte and diffuse away from the electrode. The reverse process, namely the reduction and deposition of dissolved Fe to the electrode surface is limited by mass transport due to the low concentration in the electrolyte. When an OER conditioning preceded the cyclic voltammograms, no additional peaks in the same region may be observed, since the oxidative potential prevents large-scale Fe deposition. The short reductive time window during the CV is further not able to lead to quantitative Fe deposition on the electrode surface.

For OER conditioning we can observe a slight shift, intensity increase and broadening of the NiO(OH) peaks with increasing Fe concentrations (see Figures 2C and 2D). As has been shown by Trotochaud et al. this is likely an effect of a mixed Ni_1-x_Fe_x_O(OH) species that forms, which shows an increasing shift toward lower potentials in comparison to lower Fe concentrations.^30^ The shift of the signal, especially at high temperatures, cannot be assessed, since the oxidative Ni_1-x_Fe_x_O(OH) peak disappears within the OER peak due to an increase in OER activity and thus lower OER overpotentials. Post-electrolysis CVs for both HER and OER at different Fe concentrations and temperatures can be found in Figure S3–S10 of the SI.

We determined the change in ECSA at 35°C and 55°C. The potential regions chosen were switched from 0.2–0.4 V_/RHE_ to 0.6–0.8 V_/RHE_ at higher Fe concentrations, to avoid the influence of Fe oxidation reactions at lower temperatures in the 0.2–0.4 V_/RHE_ region. The ECSA measurements at 91°C were unreliable, since in both regions faradaic reactions seem to occur. At 35°C and 55°C we can observe that for both HER and OER the capacitance at least doubles when increasing the Fe concentration from 6 to 357 μM (see Figure S11). Further, the HER capacitance is higher than the OER capacitance, indicating that Fe deposition increases the surface area.

#### Galvanostatic measurements

**Figure 3:**
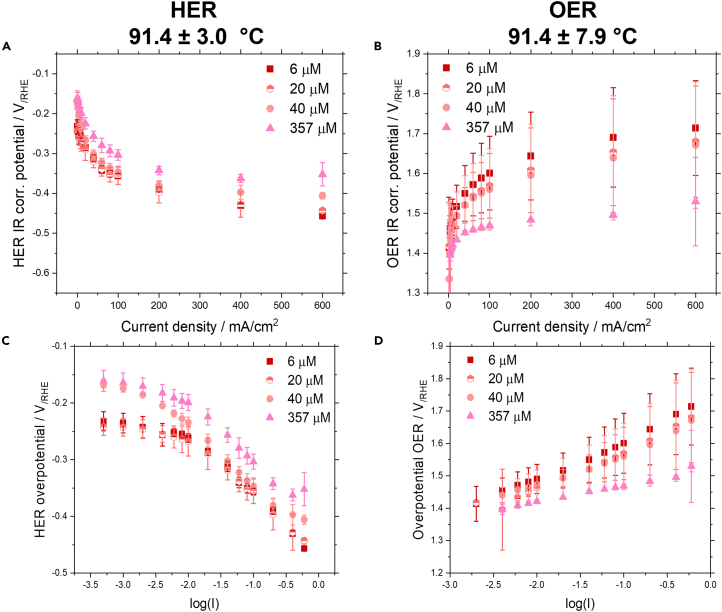
Internal resistance corrected IV curves and Tafel plots dependent on Fe concentration (A–D) IV curves for (A) internal resistance corrected HER potential and (B) internal resistance corrected OER potential and Tafel plots for (C) HER and (D) OER at 91°C for Fe electrolyte concentrations of 6, 20, 40, and 357 μM. Note that for the Tafel plots the logarithmic value of the current density was used in A/cm^2^, not mA/cm^2^. Data are represented as mean ±2 standard deviations.

To determine what effect dissolved Fe has during HER and OER, polarization (or current-voltage) curves were measured at varying current densities (see Figure 3). All experiments were conducted after an initial chronopotentiometry pretreatment at 400 mA/cm^2^ of 1.0–2.5 h to ensure that a steady state value had been reached.

We can observe that the IR-corrected HER overpotential is decreasing with an increasing Fe concentration at all temperatures (see Figures 3A; Figure S12). This is slightly unexpected, since Fe has a worse catalytic activity toward the HER in comparison to Ni.^41^^,^^42^ However, research has shown that NiFe intermixed species may be beneficial to the HER, since it prevents the formation of NiH_x_ on the cathode’s surface, which could lead to deactivation.^16^ Another reason might be the increase in surface area, leading to more active sites. For the OER we can observe a significant drop in IR-corrected half-cell potential with increasing Fe concentration at all temperatures surmounting to as much as 200 mV (see Figures 3B; Figure S14). From CV studies we have seen that with increasing electrolyte Fe concentration more mixed FeNi surface species form, especially Ni_1-x_Fe_x_O(OH), which have been shown to efficiently lead to earlier onset potentials for the OER.^30^ At lower temperatures a slight drop in IR-corrected half-cell potential can be observed for both the HER and OER with an increasing Fe concentration as well (see Figures S12 and S14).

To obtain a clearer insight into changes of the kinetics for HER and OER, we have performed Tafel slope analysis. For the HER, we can observe that the Tafel slopes may be described by bilinear slopes, changing between low and high current densities (see Figures 3C; Figure S13; Table 2). Generally, the slope is moderate (33–105 mV) at lower current densities (<20 mA/cm^2^) and steeper (92–138 mV) at high current densities (>20 mA/cm^2^). Throughout all temperature ranges we can observe that the slopes do not show a strong dependence on Fe concentration. Yet, there is a stark difference between the obtained slope values at low current densities at 35°C and 55°C in comparison to 91°C. While at lower temperatures the slope change between low and high current densities is small, at 91°C a substantial change can be observed when going from low to high current densities. Generally, the HER values for the Tafel slope are similar to values expected from literature, the only exception being the measurements conducted at 91 °C at low current densities.^43^

**Table 2:** Tafel slope values in V/dec for experiments in a 3-electrode batch cell with 30 wt. % KOH, at temperatures between 35°C and 91°C, and Fe concentrations of 6–357 μM

Temperature HER	35.4 ± 1.6°C				54.7 ± 4.5°C				91.4 ± 3.0°C			
[Fe]/μM	6	20	40	357	6	20	40	357	6	20	40	357
Linear slope< 20 mA/cm^2^	−0.096	−0.100	−0.105	−0.105	−0.091	−0.086	−0.100	−0.086	−0.033	−0.034	−0.070	−0.045
Linear slope> 20 mA/cm^2^	−0.138	−0.117	−0.104	−0.092	−0.138	−0.110	−0.101	−0.124	−0.115	−0.106	−0.095	−0.109
IR corr. potential at 8 mA/cm^2^/V_/RHE_	−0.282	−0.272	−0.258	−0.238	−0.268	−0.256	−0.243	−0.186	−0.255	−0.264	−0.228	−0.196
IR corr. potential at 600 mA/cm^2^/V_/RHE_	−0.549	−0.476	−0.413	−0.431	−0.518	−0.460	−0.412	−0.419	−0.457	−0.443	−0.406	−0.352

Temperature OER	**34.7 ± 2.0°C**				**55.1 ± 2.3°C**				**91.4 ± 7.9°C**			
[Fe]/μM	**6**	**20**	**40**	**357**	**6**	**20**	**40**	**357**	**6**	**20**	**40**	**357**
Linear slope< 20 mA/cm^2^	0.057	0.042	0.037	0.034	0.044	0.035	0.033	0.031	0.101	0.113	0.077	0.053
Linear slope> 20 mA/cm^2^	0.079	0.084	0.058	0.056	0.066	0.040	0.036	0.029	0.136	0.129	0.119	0.057
IR corr. potential at 8 mA/cm^2^/V_/RHE_	1.552	1.533	1.513	1.497	1.505	1.479	1.466	1.459	1.481	1.452	1.464	1.415
IR corr. potential at 600 mA/cm^2^/V_/RHE_	1.709	1.687	n.a.	1.601	1.623	n.a.	1.535	1.519	1.714	1.680	1.671	1.529

Bilinear slope fitting was conducted, the data was fitted in the low current density regime (0.5–20 mA/cm^2^) and the high current density regime (20–800 mA/cm^2^).

By examining the IR corrected potentials at low and high current densities of 8 and 600 mA/cm^2^, respectively, we are further able to evaluate the catalytic activity of the electrode. Here, we can observe throughout all temperatures ranges that with an increasing Fe concentration the potential is decreasing, suggesting that the activating effect of the iron results from an increase in the exchange current density of the HER.

Similarly, to the HER, the Tafel slopes obtained for the OER may be described by bilinear fitting (see Figures 3D; Figure S15; Table 2). Here, we can observe a significant decrease in slopes with increasing Fe concentrations throughout all temperature ranges. This showcases the influence of electrolyte Fe on kinetics. The slopes for the OER (31–79 mV) at moderate temperatures are lower in comparison to the HER, as is expected from literature.^43^ Interestingly, increasing the temperature to 91°C leads to an increase in the slopes at low and high current densities.

The catalytic activity of the OER electrode is also assessed using the internal resistance corrected potential at 8 and 600 mA/cm^2^. Here, we observe that the potential is decreasing with an increasing Fe concentration, pointing toward a better catalytic activity of the electrode toward OER. This is not unexpected, since it has been shown in literature that increasing the Fe doping in the electrolyte leads to an earlier onset potential for OER. This is likely connected to the formation of reactive Ni_1-x_Fe_x_O(OH) species on the anodes surface, with higher Fe concentrations supporting the formation of this reactive species. The activating effect of Fe seems to be influenced by the temperature as well: a higher temperature leads to a stronger activation at higher Fe concentrations in comparison to lower temperatures.

### Flow cell studies

Analogous to the three-electrode experiments, the studies in the flow cell were carried out with 30 wt. % KOH with an addition of 0, 25, 50, and 500 μM of Fe in the form of Fe_2_(SO_4_)_3_ hydrate. At added Fe concentrations of 50 μM and higher it could be observed that some precipitated Fe(OH)_3_ was observed in the gas liquid separator for the duration of the experiments, while at 25 μM the particles quickly disappeared.

#### ICP measurements

To determine the real concentration of mobile Fe in the electrolyte, ICP-OES measurements were conducted pre and post electrolysis (see Table 3).

**Table 3:** Fe concentration in the electrolyte pre and post electrolysis as determined by ICP-OES

[Fe] sample	/μM	/in ppm (H_2_O)
0 μM Fe pre electrolysis	13.4 ± 1.6	0.24
0 μM Fe post electrolysis	17.3 ± 2.2	0.31
25 μM Fe pre electrolysis	44.4 ± 0.3	0.80
25 μM Fe post electrolysis	58.1 ± 1.1	1.05
50 μM Fe pre electrolysis	55.1 ± 6.3	0.99
50 μM Fe post electrolysis	45.6 ± 5.2	0.82
500 μM Fe pre electrolysis	548.5 ± 60.8	9.87
500 μM Fe post electrolysis	274.5 ± 30.4	4.94

Pre electrolysis experiments were conducted before each experimental batch and post electrolysis experiments after conclusion of the entire experimental batch (experimental batch incl. experiments at all temperatures).

Compared to the three-electrode results, we can observe a higher Fe concentration in the electrolyte where no Fe was added. This can be traced back to a different batch of KOH pellets being used for the experiments. The experiments conducted without addition of Fe showed a slight increase of the Fe concentration post electrolysis. Here, we must note that electrolyte samples taken had to be diluted 1:100 to not damage the ICP-OES with high K concentrations. This means we are operating closer to the lower detection limit of the ICP-OES, which would explain the slight variance of values. The Fe concentration for the 25 μM experiments is increased post-electrolysis in comparison to pre-electrolysis. Prior to the 25 μM experiments, experiments at higher Fe concentrations were conducted in the same electrolysis setup and as such remaining traces of Fe might have increased the total Fe concentration in the electrolyte. When the Fe concentration exceeds the solubility limit (in our case starting at 55.1 μM), we can observe a decrease in concentration post electrolysis.

#### Galvanostatic measurements

**Figure 4:**
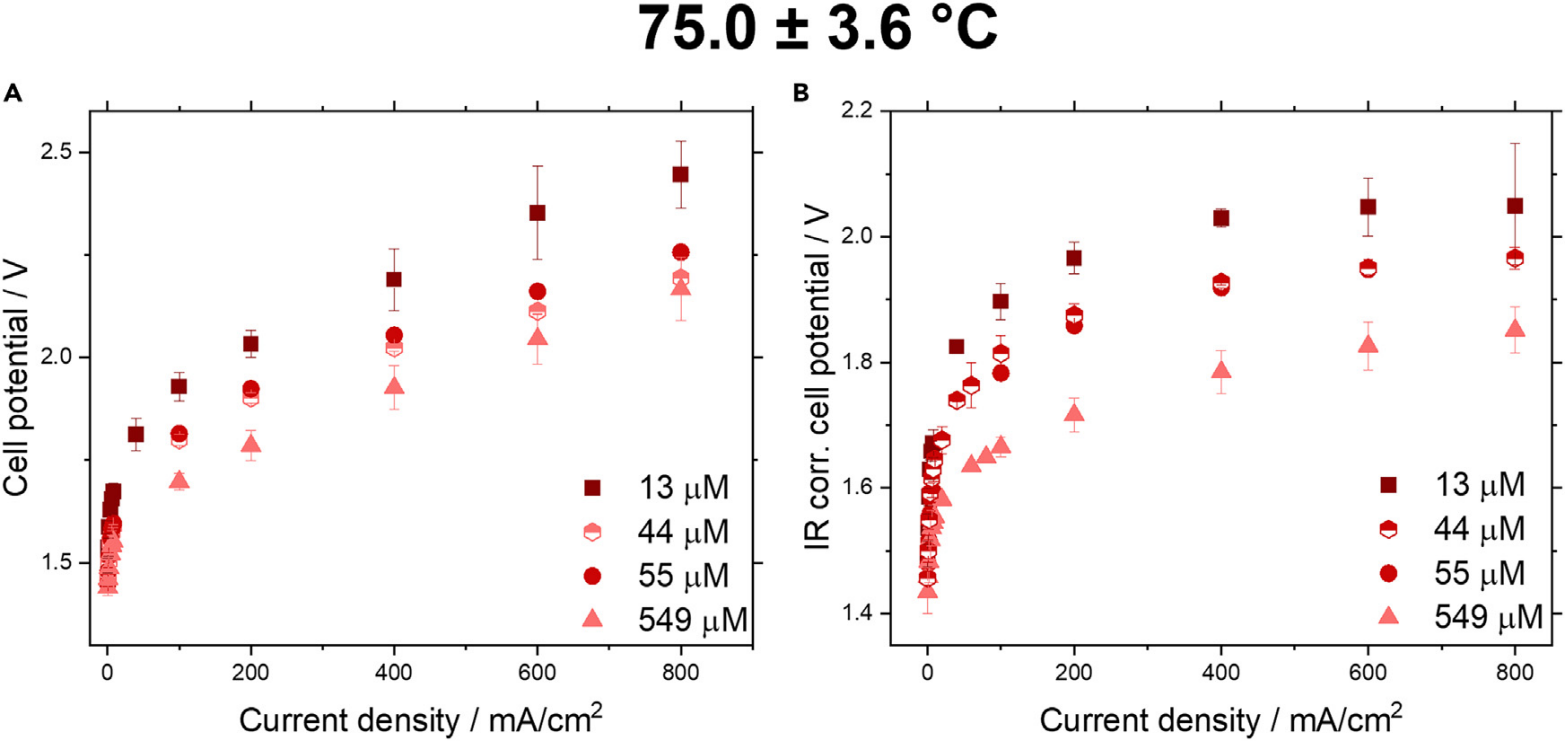
IV curves without and with internal resistance correction dependent on Fe concentration (A and B) (A) IV curves determined from chronopotentiometry in a flow cell with 30 wt. % KOH with Fe conc. of 13, 44, 55, and 549 μM at 75.0 ± 3.6°C and (B) IV curves with internal resistance corrected potentials for the same electrolyte compositions at 75.0 ± 3.6°C. Data are represented as mean ±2 standard deviations.

The obtained IV curves for the cell potential after pretreatment for the different Fe concentrations at 75.0 ± 3.6°C can be seen in Figure 4A. A drop in cell potential can be seen with increasing Fe concentration. To assess the influence of the iron concentration on the overpotentials the ohmic resistance was determined via EIS at the same current densities, and subtracted from the cell potential to obtain the IR corrected cell potential (see Figure 4B). Nyquist plots and Bode plots can be found in the SI in Figures S18 and S19. The ohmic resistance at 75.0 ± 3.6°C is also shown in Figure S20, which shows that the ohmic resistance stays largely independent of the iron concentration. The ohmic resistance at other temperatures can be found in Figure S17. We can observe that with increasing Fe concentration the cell potential keeps decreasing. This contrasts with the work of Chung et al., which showed no further enhancement of iron above 0.1 ppm.^31^ This continuous enhancement of the activity is quite surprising, especially at 549 μM of Fe, since complete saturation of the electrolyte solution occurs. Additionally, the enhancing effect of Fe can be observed throughout all current density regions and temperatures ranges (see Figure S16). To ensure that the increased activity cannot be solely connected to Fe oxidation/reduction, we calculated the limiting current density based on the Fe concentration for a parallel plate flow cell. Here, we found that the limiting current density for a Fe concentration of 549 μM is 38 μA/cm^2^ and can thus be neglected (see Table S3).

**Figure 5:**
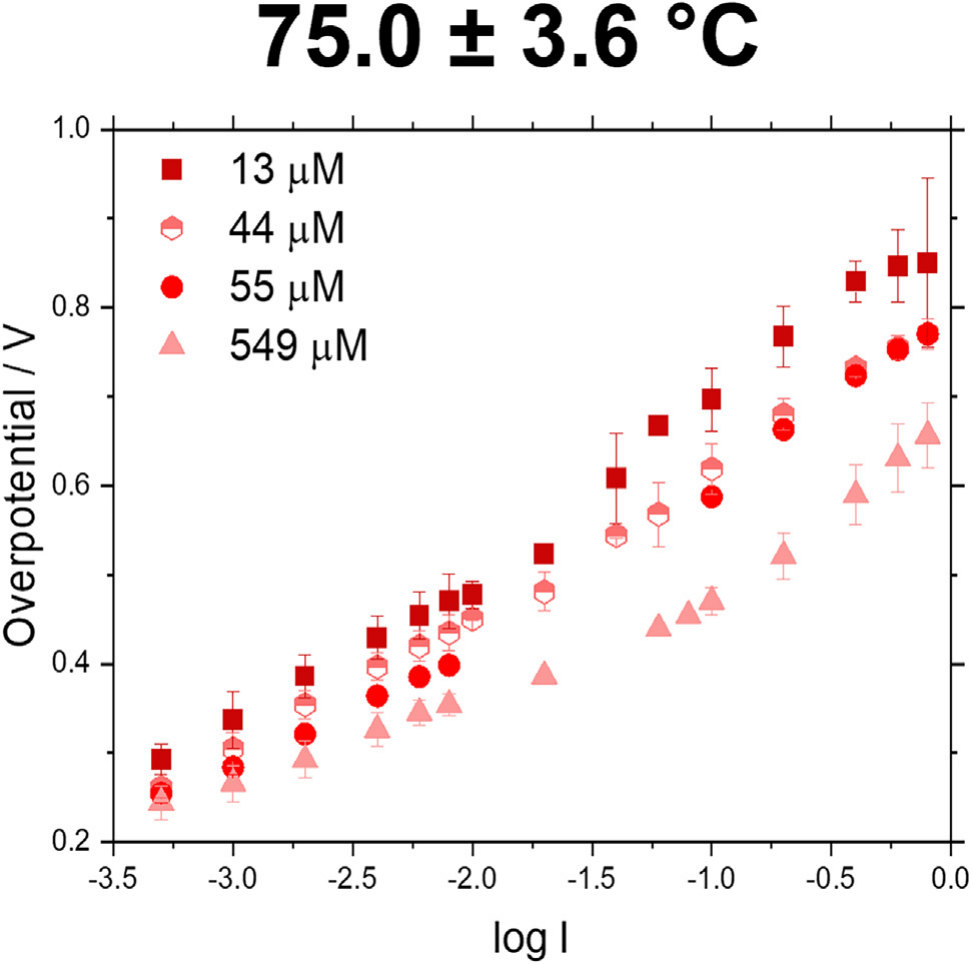
Tafel slopes at 75.0 ± 3.6°C for the flow cell with 30 wt. % KOH with Fe concentrations of 13, 44, 55, and 549 μM Note that for the Tafel plots the logarithmic value of the current density was used in A/cm^2^, not mA/cm^2^. Data are represented as mean ±2 standard deviations.

To obtain a clearer picture on the kinetics that occur in the flow cell, Tafel slope analysis was performed. For the flow cell measurements with only two electrodes, the Tafel slope obtained will always be a combination of both electrodes. As can be seen in Figure 5, Figure S21, and Table 4 the Tafel slopes can be described by a bilinear slope. The slope change occurs usually in the range of 20 mA/cm^2^ (∼log(I) = −1.70). The bilinear fitting reveals that only in the low current density regime (0.5–20 mA/cm^2^) a significant improvement in the Tafel slope can be observed with increasing Fe concentration, while in the high current density regime (20–800 mA/cm^2^) the Tafel slope does not change significantly. Similarly, with increasing temperature a reduction of the slope can be seen in the low current density regime, however, not in the high current density regime.

**Table 4:** Tafel slope values in V/dec for experiments in a flow cell with 30 wt. % KOH, at temperatures between 21°C and 75°C, and Fe concentrations of 13–549 μM

Temperature	21.0 ± 1.9°C				46.9 ± 2.1°C				75.0 ± 3.6°C			
[Fe]/μM	13	44	55	549	13	44	55	549	13	44	55	549
Linear slope< 20 mA/cm^2^	0.160	0.149	0.143	0.130	0.154	0.152	0.135	0.096	0.144	0.140	0.123	0.095
Linear slope> 20 mA/cm^2^	0.369	0.163	0.164	0.193	0.200	0.166	0.149	0.217	0.209	0.184	0.202	0.210
IR corr. potential at 8 mA/cm^2^/V	1.869	1.789	1.796	1.747	1.811	1.732	1.686	1.614	1.671	1.630	1.594	1.545
IR corr. potential at 800 mA/cm^2^/V	2.357	2.243	2.154	2.120	2.171	2.064	2.011	1.988	2.049	1.995	1.966	1.851

Bilinear slope fitting was conducted, the data was fitted in the low current density regime (0.5–20 mA/cm^2^) and the high current density regime (20–800 mA/cm^2^).

When comparing IR corrected potentials at 8 and 800 mA/cm^2^ (see Table 4), we can observe throughout all experiments that with increasing Fe concentration and temperature a decrease in IR corrected potential can be achieved.

#### SEM/EDX measurements

The change of the elemental surface composition on the working electrode was assessed using SEM/EDX analysis (see Table 5). The main elements that were assessed via EDX were carbon, oxygen, potassium, iron, and nickel.

**Table 5:** Weight percentage of elements present on the surface of the working electrodes used either for HER or OER in the three-electrode cell and for the cathode and anode used in the flow cell pre- and post-electrolysis at 30 wt. % KOH with varying Fe concentrations

Sample	Elemental weight percentage three electrode cell					Fe/Ni ratio
	C	O	K	Fe	Ni	
*Pristine electrode*	5.9	1.2	0.0	0.0	92.9	0.000
*HER 6 μM Fe*	9.4	26.8	24.5	2.0	32.8	0.060
*HER 20 μM Fe*	5.5	11.2	5.6	10.7	62.4	0.171
*HER 40 μM Fe*	5.1	24.3	15.5	14.1	38.5	0.366
*HER 357 μM Fe*	2.8	29.2	45.5	5.2	7.4	0.696
*OER 6 μM Fe*	7.5	14.8	5.8	0.3	70.0	0.004
*OER 20 μM Fe*	10.8	19.5	21.9	0.4	41.5	0.008
*OER 40 μM Fe*	9.3	30.4	37.8	12.7	7.2	1.767
*OER 357 μM Fe*	3.1	23.4	16.3	4.3	58.6	0.083

**Elemental weight percentage flow cell electrodes**						

*Pristine electrode*	0.6	1.8	0.0	2.1	95.5	0.022
*Cathode 13 μM Fe*	0.6	5.5	0.0	5.5	88.4	0.062
*Cathode 44 μM Fe*	7.4	15.0	9.3	16.1	50.0	0.322
*Cathode 55 μM Fe*	7.0	10.9	9.9	37.7	34.0	1.109
*Cathode 549 μM Fe*	0.0	3.8	0.9	31.5	63.8	0.494
*Anode 13 μM Fe*	0.1	5.8	3.4	0.7	90.0	0.008
*Anode 44 μM Fe*	1.1	3.3	0.0	1.9	93.7	0.020
*Anode 55 μM Fe*	8.2	13.0	10.2	0.4	68.2	0.006
*Anode 549 μM Fe*	8.5	5.6	2.5	1.0	82.3	0.012

The pristine electrodes used in the three-electrode study were essentially free of Fe, with mainly Ni, C, and O being detected. The O probably originates from Ni oxidized by air, while the C likely originates from environmental contamination of the electrode by carbon containing organics.

On the used electrodes, K and O can often be found co-deposited with each other and KOH crystal formation in those areas can be observed using SEM. The samples were not washed prior to analysis, since the formed layers on the electrode might be so fragile that they could be easily washed away. Surprisingly, the O content on the electrode after OER appears to not be significantly higher than for the cathode, in spite of the highly oxidative conditions. Since the electrodes were measured up to several days post electrolysis, an air mediated oxidation of the surface could have occurred in the meantime.

When the working electrode was employed for HER, an increasing Fe to Ni ratio can be observed with an increasing electrolyte Fe concentration. Generally, we can thus conclude that more Fe is deposited on the electrodes surface with an increasing Fe electrolyte concentration.

Differently to the HER, when the working electrode is employed for OER, we can see that the surface Fe to Ni ratio is rather invariant to the electrolyte Fe concentration. This means there is no quantitative surface deposition of Fe that takes place. At 40 μM electrolyte Fe for OER we see a stark increase in the Fe to Ni ratio. We suspect, that this might be caused by prolonged water evaporation overnight and electrolyte drying up on the electrode, which contains Fe. As such that point might be considered an outlier.

Analogous to the three-electrode measurements, the main elements that were assessed via EDX for the electrodes used in the flow cell were carbon, oxygen, potassium, iron, and nickel (see Figures S22–S25). For pristine electrode material made from Ni201 plates, already 2 wt. % Fe can be found, likely a result of the manufacturing process. The electrode appears also to be slightly oxidized, since up to 1.8 wt. % O can be detected.

The C likely originates from environmental contamination. The Fe weight percentage on the anodes does not seem to increase quantitatively with increasing Fe concentration in the electrolyte, at least within the detection limits of the EDX. Generally, between 0.4 and 1.9 wt. % of Fe are detected on the anode, never exceeding the maximum amount of Fe detected for a pristine electrode. This would suggest that no significant Fe deposition occurs on the surface of the anode. This is in contrast to the cathode: here, the amount of deposited Fe increases with increasing electrolyte Fe concentration up to 55 μM. The amount of cathode surface deposited Fe is similar between 55 and 549 μM Fe, suggesting that a maximum of surface deposited Fe may be reached on the cathode. This is also reflected by the increasing Fe to Ni ratio on the cathode. Interestingly, the Fe to Ni ratio on the flow cell cathode is increasing faster with increasing Fe concentration than the Fe to Ni ratio on the HER electrode which was used during three-electrode measurements. There are two potential reasons for this: (1) the flow promotes mass transport of Fe to the cathode or (2) since the potential for the cathode was never changed from reductive to oxidative conditions (that means no CVs were measured at the start and end of an experimental row), the deposited Fe was never dissolved again. This would lead to higher Fe to Ni ratios on the surface.

**Figure 6:**
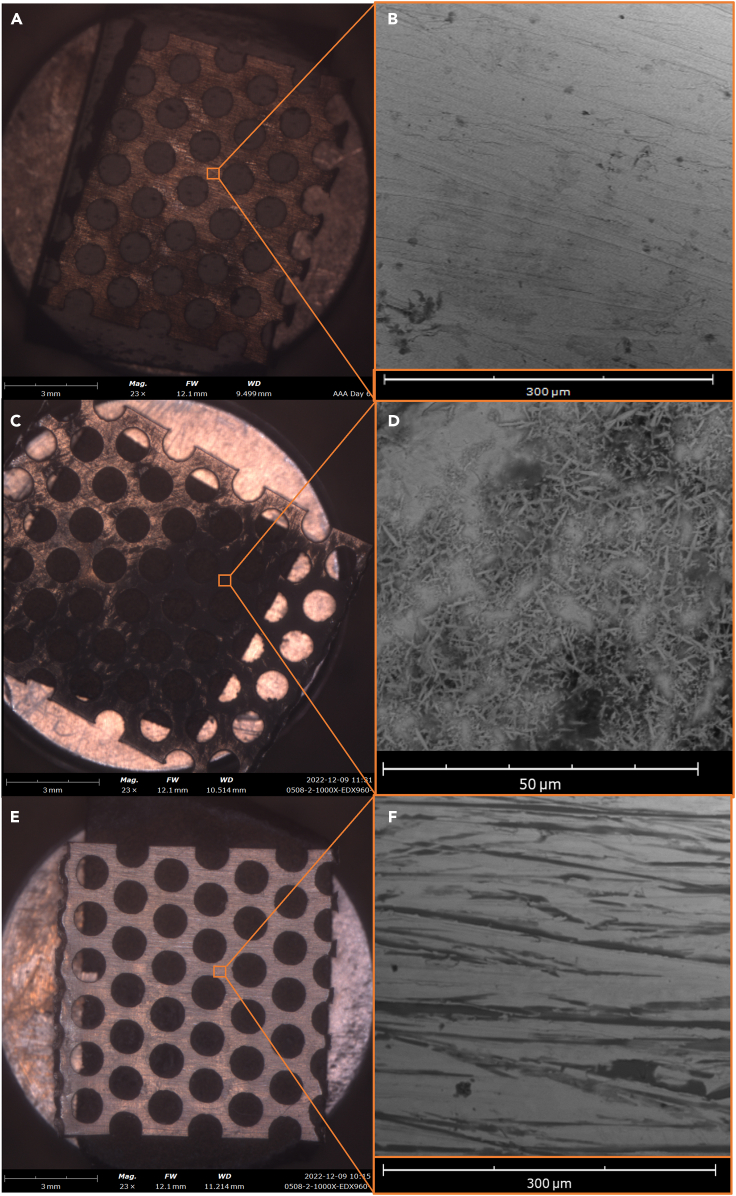
Optical and SEM images of electrodes post electrolysis (A and B) Optical image of cathode and SEM image of the cathode’s surface post-electrolysis with 13 μM of electrolyte Fe showing only slight indications of dendrite formation. (C and D) optical image of cathode and SEM image of Fe deposited dendrite structures on the cathode’s surface post electrolysis with 55 μM of electrolyte Fe. (E and F) optical image of anode and SEM image of the anode’s surface post electrolysis with 55 μM of electrolyte Fe showing the lack of dendrite structures (the black streaks are dried KOH residues).

The Fe particles that form on the cathode surface have a dendritic shape, forming black needle-like crystals, a few μm in size (see Figures 6C and 6D). First indications of dendrite formation can already be seen at low Fe concentrations (see Figures 6A and 6B), however, they are more prevalent at higher Fe concentrations (see Figures 6C and 6D). The black color (see Figure 6C) suggests that the formed dendrites are made of metallic Fe, which deposited due to reduction from electrolyte Fe, analogous to Brossard’s observations.^18^ Similar processes have been observed in the past on an industrial scale, e.g., for chlor-alkali electrolysis.^19^ Due to the aforementioned impurities of Fe in the electrolyte, over time large dendrites up to several tens of μm would form on the cathode. In comparison, in our experiment, the time span of the experiments was short and as such larger dendrites did not form on the surface. While the dendrites might block the Ni surface, they are benefitting the HER as determined in the three-electrode studies, since they result in a 4-fold higher capacitance (and hence ECSA) than the pristine perforated Ni plate. The catalytic activity between Fe and Ni for HER also are largely similar, which is why in this experiment no negative impact of dendrite formation could be observed. On the anode’s surface no indication of Fe deposition was observed (see Figures 6E and 6F).

#### XPS measurements

**Figure 7:**
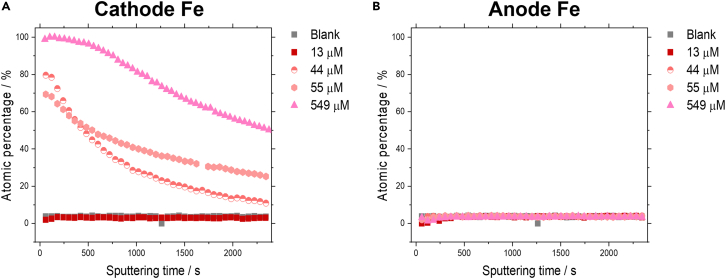
Electrode depth profiles as determined by XPS sputtering (A and B) Atomic percentages of Fe on the (A) cathode and (B) anode dependent on ion gun sputtering time as measured by XPS.

To further determine the chemical surface composition of the flow cell electrodes, XPS was employed. We sputtered into the surface using an ion gun, to observe the thickness of the surface deposited Fe (see Figures S26–S30 for XPS spectra). A pristine electrode blank was measured to differentiate the electrode surface composition between pre and post electrolysis. The high-resolution XPS spectra exhibit the main Fe 2p 3/2 peak at 705.6 eV, which is an indication of the presence of Fe(0). Additionally, structures are observed between 709 and 716 eV, which could be an indication of the presence of FeOOH species, however a significant overlap with the Ni LMM line (elements within the 2p block often eject Auger electrons; these peaks are commonly labeled as LMM transitions) is present as seen from the pristine electrode XPS measurements (Figure S26) so no clear assignment can be made. No additional satellite structures are observed between 716 and 720, providing an indication that no iron is present in the +2 or +3 oxidation state.^44^ However, since oxidation states might vary significantly during electrolysis, the main focus was set on determining the Fe to Ni ratio as a function of the layer depth. On the cathode we can see that with increasing Fe concentration the amount of surface deposited Fe increases and also the thickness of the surface layer (see Figure 7). At 549 μM the surface deposited Fe reaches at least up to 1.85 μm in thickness as determined by the sputtering time of the ion beam with an approximate etching rate of 0.37 nm/s compared to Ta_2_O_5_ (after 5000 s of sputtering still ∼30 at% of Fe were detected). Intriguingly, there is no significant difference between a blank electrode and a cathode used with 13 μM of Fe (no additional Fe added to the electrolyte). This would indicate that additional Fe, which is added to the electrolyte, seems to promote deposition on the cathode’s surface. Interestingly, we do not see a change in Fe content in the surface composition of the anode post electrolysis with increasing electrolyte Fe concentration.

## Discussion

Throughout our studies, we were able to assess that Fe in the electrolyte significantly impacts the performance of an AWE cell. The surface composition of the cathode and anode during electrolysis changes significantly, as evident by the cyclic voltammetry, SEM/EDX, and XPS measurements.

On the cathode hydrogen evolution and iron reduction takes place, resulting in the formation of elemental Fe, which forms large, black dendrites on the cathodes surface over the course of several hours. However, the Fe dendrites quickly dissolve or disperse again to form Fe hydroxides, when an oxidative potential is applied, as seen in consecutive full CV measurements. The thickness of the surface deposited Fe increases with increasing Fe concentration as seen with XPS. Through ECSA measurements we could observe that the formation of Fe dendrites additionally coincides with an increase of surface area. Those trends were found to be more pronounced with higher electrolyte Fe concentration. While higher Fe concentrations did not lead to a significant drop in the Tafel slope, we could assess by the decreasing IR corrected potential, that the catalytic activity of a NiFe co-doped surface is increased, likely connected to the increased surface area and the prevention of NiH_x_ and thus deactivation of the surface.

On the anode we see a change in surface composition as the Fe and Ni together form mixed NiFe hydroxide species and at more oxidative potentials Ni_1-x_Fe_x_O(OH) species. Using XPS, we saw that no significant reconstruction of the Ni surface occurred, giving rise to the assumption that a potential NiFe layer on the anode is only a few atomic layers thick. Still we could find an increase in capacitance for OER electrodes. In literature the idea of extremely thin Fe layers in the outer Helmholtz plane has been discussed before and also the loose Fe incorporation only during polarization has been suggested.^45^^,^^46^ After polarization, the Fe is then released back to the electrolyte. As has been shown the formation of Ni_1-x_Fe_x_O(OH) species seems to benefit the OER, as is evident from a decreasing Tafel slope and OER onset potential.^30^ Increasing the Fe concentration hence directly benefits the OER, even though no quantitative surface reconstruction was observed. However, at low electrolyte Fe concentrations we observed that the Fe concentration was always increased post electrolysis in almost entirely Fe free setups. Since Ni201 electrodes may contain up to 2% of Fe in their pristine form, this means that there is a chance that Fe leaches from the electrode to the electrolyte, leading to Fe depletion on the electrode. Here, high electrolyte Fe concentrations can also serve as a healing mechanism to avoid Fe depletion on the anode, improving their overall performance.

When applied to a flow cell, we can see the combined effect that Fe has on HER and OER. It seems to us that the impact of Fe can especially be seen at those high current densities, which are not reached in a CV. Even above the solubility limit of Fe hydroxides, we can observe a non-negligible increase in the performance of the cell. Hereby, we could see that those improvements with higher Fe concentrations were of similar magnitudes for all temperatures. Within our studies utilizing a Zero-gap configuration, we achieved current densities of 400 mA/cm^2^ at a (non-IR-corrected) cell potential of 1.93 V at 549 μM electrolyte Fe and a temperature of 75°C. This is already comparable to typical conventional alkaline electrolyzers that operate at 1.8–2 V achieving current densities of up to 300 mA/cm^2^.^47^ Further improvements to the cell potential may be expected, if thinner diaphragms (Zirfon UTP Perl 220 instead of Zirfon UTP Perl 500) are used, contact resistances are minimized and if activated cathodes with high surface areas are employed, e.g., Raney-Ni.^48^^,^^49^

**Figure 8:**
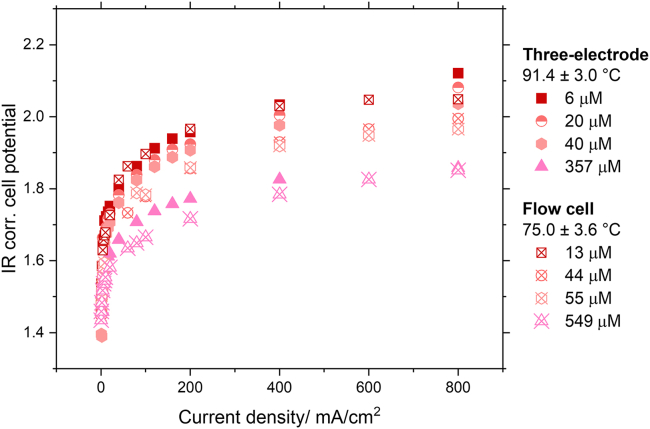
Comparison of IR corrected potentials for the combined HER and OER obtained using the three-electrode cell and the IR corrected cell potentials obtained using the flow cell Note that here for the three-electrode cell electrodes a geometric surface area of 1 cm^2^ is assumed (equivalent to the front area of the electrode).

Overall, we can conclude that doping the electrolyte with trace amounts of Fe in the ppm range increases electrolyzer performance, when using conventional Ni electrodes. To compare the performance of the three-electrode cell with the flow cell, we used the IR-corrected IV curves. When comparing the IR corrected cell potentials of the flow cell with the combined IR corrected potentials for HER and OER, we can observe that the flow cell and the three-electrode cell electrodes experience a similar enhancing effect by the electrolyte Fe (see Figure 8). Minor differences occur, with the flow cell showing slightly lower potentials. This is counterintuitive, since the three-electrode cell was operated at higher temperatures and as such the performance should be better. We suspect that there are two main reasons for this: (1) the flow of the flow cell serves to overcome Fe mass-transfer limitations. This allows Fe to be faster incorporated into the electrodes surface, making the electrodes more active. (2) In the flow cell the Fe mass to electrode surface area ratio is higher. The flow cell takes up a volume of up to 1.2 L of electrolyte, while the three-electrode cell only has a total volume of around 0.2 L. This means that at the same Fe concentration, quantitatively more Fe is available to be deposited on the electrodes.

### Limitations of this study

As we have established in our observations, the higher the amount of available electrolyte Fe to deposit on the electrodes, the better the overall performance. Within the scope of our study, we were not yet able to determine a maximum threshold for this trend. Additional limitations apply to our study. Long term experiments over the course of 100+ or even 1000+ hours were not conducted (maximum total experimental time being 16 h of flow cell operation with breaks). If dendrite growth on the electrodes becomes too dominant, a decrease in performance could occur. Similarly, performing accelerated stress testing would help to determine the stability of the catalyst and electrochemical operations. As was shown in the CV measurements, the deposited Fe quickly dissolves when exposed to oxidizing potentials. Furthermore, the reason for the performance increase at high Fe concentrations exceeding the Fe hydroxide solubility limit is not yet clear. Colloid formation of Fe can be hypothesized to be a potential cause. Operando techniques, like *in situ* XPS, would further help unravel which active Fe surface species are formed during electrolysis operation to help understand the catalytic effect of Fe on OER and HER better.

Within our study we performed experiments with a three-electrode cell and a flow cell to determine the influence of electrolyte Fe on AWE using Ni electrodes. In the three-electrode cell Fe promotes both HER and OER by lowering the overpotential of each reaction by at least 100 mV at high current densities when increasing the Fe concentration of the electrolyte from 6 to 357 μM. Similarly, the overpotential of the zero-gap flow cell was decreased by 200 mV when increasing the Fe concentration from 13 to 549 μM. HER mainly benefits from the formation of Fe dendrite layers that form on the surface of the electrodes, which prevents NiH_x_ formation and increases the overall active area. The OER probably benefits from the formation of thin layers of mixed Ni/Fe oxyhydroxides which form on the surface. This leads to better catalytic activity and a reduction of the Tafel slopes.

## STAR★Methods

### Key resources table

**Table:** 

REAGENT or RESOURCE	SOURCE	IDENTIFIER
**Chemicals, peptides, and recombinant proteins**		

Ni-201 perforated plates	Metel B.V.	13010116310000
KOH	VWR chemicals	26669.460
Fe_2_(SO_4_)_3_ hydrate	Fluka Analytical	7664-93-9

**Deposited data**		

Measurement data	This paper	Publicly available under: https://data.mendeley.com/datasets/8dd5756cxm/1
Used code for data analysis	This paper	Publicly available under: https://data.mendeley.com/datasets/8dd5756cxm/1

### Resource availability

#### Lead contact

Further information and requests for +resources and reagents should be directed to and will be fulfilled by the lead contact, Maximilian Demnitz (m.demnitz@tue.nl).

#### Materials availability

This study did not generate new unique reagents.

#### Data and code availability

(1) Models have been deposited with Mendeley datasets and are publicly available as the date of publication under https://data.mendeley.com/datasets/8dd5756cxm/1.
(2) All raw and analyzed data have been deposited with Mendeley datasets and are publicly available as the date of publication under https://data.mendeley.com/datasets/8dd5756cxm/1.
(3) Any additional information required to reanalyze the data reported in this paper is available from the lead contact upon request.

### Method details

#### Electrolyte preparation

For experiments a 30 wt. % KOH electrolyte (≥88% purity; VWR chemicals) was prepared. The KOH pellets are hygroscopic, resulting in up to 12% H_2_O. To dope the electrolyte 12.5, 25, or 250 μM of Fe_2_(SO_4_)_3_ hydrate (≥76% assay, ≤24% water; Fluka Analytical) was added, corresponding to electrolyte Fe concentrations of 25, 50, and 500 μM, respectively.

#### Three electrode studies

For 3-electrode measurements we used a Ni-201 plate (Metel B.V.) with a total front and back area of 2 cm^2^ with round perforations (d = 1 mm) and an open area of 40% as a working electrode and a folded Ni-mesh (100 mesh woven from Ø = 0.1 mm wire, Thermo Scientific Chemicals) as a counter electrode. Both, electrodes and mesh, were ultrasonicated in a 1:1 iso-propanol/water solution to clean the electrodes. For every experiment the working electrode was either used for HER or OER at one specific Fe concentration. After each experiment, the working electrode and counter electrode were exchanged for pristine electrodes. The potential of the working electrode was measured against a reversible hydrogen electrode (RHE) as reference (Hydroflex, Gaskatel). Within the cell the reference electrode was placed within a small compartment which was connected to the main cell via a luggin capillary. The potential or current were applied using a potentiostat (Compactstat.h06125 1.25A/6V, Ivium Technologies). The electrodes were held in place within the 3-electrode cell using tantalum wire clips (Redox.me). The cell was filled with 30 wt. % KOH electrolyte containing a specific Fe concentration. The solution was stirred using a magnetic stirrer in combination with a magnetic heating plate. The temperature in the cell was monitored via two thermocouples in the main chamber (connected to the potentiostat) and in the reference electrode compartment (connected to a data logger). This resulted in electrolyte temperatures of 35.4 ± 1.6, 54.7 ± 4.5, and 91.4 ± 3.0°C (¯T ± 2σ; taken from all measurements) used for HER and temperatures of 34.7 ± 2.0, 55.1 ± 2.3, and 91.4 ± 7.9°C (¯T ± 2σ; taken from all measurements) used for OER.

As a first step full cyclic voltammetry (between −0.35 and 1.6 V_/RHE_) runs were conducted. The full CVs were recorded at the start and end of the experimental set at the same temperature or before the start of the following experimental set at higher temperature (experimental set at 35°C → experimental set at 55°C → experimental set at 91°C; see Figures 1 and S3–S10). Please note that due to errors in the potentiostat software some of the end full CV measurements were not conducted. However, all other measurements were conducted as intended. However, by extend, the next start full CV at the following higher temperature can be taken as replacement for the missing end full CV at the same temperature (also see Table S1). After the first full CV the working electrode was then conditioned at 400 mA/cm^2^ (positive current for OER; negative for HER) for 1 h and thereafter the ECSA was determined by performing cyclic voltammetry (CV) in the non-faradaic range of 0.2–0.4 V_/RHE_ or 0.6–0.8 V_/RHE_ at scan rates between 10 and 400 mV/s. Following, chronopotentiometry (CP) and electrochemical impedance spectroscopy (EIS) measurements were conducted from 1 to 600 mA/cm^2^ for the three-electrode experiments. For a precise description of the individual measurement steps, we refer to Table S1 in the Supplementary Information (SI). The individual points of the obtained IV curves were determined from the average last 10 s of the chronopotentiometry measurements. The ohmic resistances at each current density (see Figures S13A–S13C and S15A–S15C) for HER and OER were determined via EIS and subtracted from the corresponding half-cell potentials to obtain the IR-corrected values. For each temperature and Fe concentration this procedure was carried out at least three times, to obtain multiple measurements for each point. For each measured point the error bar was determined by using two times the standard deviation of the dataset.

#### Flow cell studies

The experiments were carried out in a 3D-printed flow cell (in-house design) made of polypropylene (PP), where two Ni-201 electrodes are used as a cathode and anode, respectively (see Figure S2). Within this study we used two versions of the flow cell: experiments at 0, 50, and 500 μM electrolyte Fe were conducted in an older cell, while experiments at 25 μM electrolyte Fe were conducted in a new iteration of the PP flow cell (with the same geometry as old cell). Almost all the individual components of the electrolyser setup (see Figure S1 for the setup scheme), that are in contact with the electrolyte, are free of Fe. Notable exceptions are a centrifugal pump with Hastelloy head and two 3-way valves made of stainless steel to drain the system. The Ni electrodes are 0.95 × 3.20 cm^2^ in size with round perforations (d = 1 mm) leading to an open area of ∼40% and an active geometric area of 2.5 cm^2^. The current to the electrodes is supplied and collected via stainless steel screws. A potentiostat (Vertex.10A, Ivium Technologies) is used, which is connected to the screws via crocodile clips in a 2-terminal configuration. The individual half-cells of the flow cells are connected to two glass gas-liquid separators via PP tubing. The outgoing catholyte and anolyte are mixed again before entering a centrifugal pump (Model DGH-57, Tuthill), which pumps the electrolyte back to the flow cell at a flow rate of 4 mL/s. The gas in the gas liquid separator is diluted via a continuous N_2_ stream and led to a washer with deionized water. The temperature of the setup is controlled via a Brinkmann MGW Lauda C6 thermostat (with Brinkmann MGW Lauda R22 and CS control units), which is connected to the gas-liquid separators. The actual temperature of the electrolyte is monitored via thermocouples located at the inlet of the flow cell. The thermostat temperature for the individual experiment runs was set to 20, 50, and 85°C. This resulted in electrolyte inlet temperatures of 21.0 ± 1.9, 46.9 ± 2.1, and 75.0 ± 3.6°C (¯T ± 2σ), respectively. The temperatures were lower than for the 3-electrode cell, since heat losses occur within the system.

The electrolyte was then added to the setup via the gas liquid separators. The Fe concentration in the electrolyte was determined pre and post electrolysis using ICP-OES (Inductively Coupled Plasma – Optical Emission Spectroscopy; iCAP PRO ICP-OES; Thermo Scientific). The cathode and anode were also analyzed pre and post analysis using SEM/EDX (Scanning Electron Microscopy/Energy Dispersive X-ray spectroscopy; Phenom ProX G6 Desktop SEM; Thermo Scientific) as well as XPS (X-ray Photoelectron Spectroscopy). Note that for the EDX analysis at 15 keV the surface bulk penetration depth for Ni and Fe is at maximum 8–10 μm^50^

The electrodes were further investigated using conventional ultrahigh vacuum XPS (Thermo Scientific K-Alpha, equipped with an Al anode (Al K_α_ = 1,486.68 eV) monochromatized X-ray source). Samples were prepared by placing a cut out section of the spend electrode onto double sided carbon tape. Wide-range survey spectra were recorded using a 200 eV pass energy, while high-resolution core level spectra were measured using a 50 eV pass energy. The background pressure inside the analysis chamber was kept below 8 · 10^−8^ mbar. All spectra were recorded using the flood gun (low energy Ar^+^ ions) to account for surface charging.

Atomic surface ratios were estimated making use of the atomic sensitivity factors and the subtraction of the Shirley-type background using the CasaXPS software version 2.3.23 rev 1.2K. Energy referencing was performed using the adventitious carbon peak at a reference binding energy of 284.5 eV.

In order to allow for investigation below the surface of the electrode, XPS depth profiling was used through etching under ultrahigh vacuum inside the XPS apparatus to collect C1s, O1s, Fe 2p and Ni2p high-resolution spectra. The depth profiling was performed by etching the surface using an Ar ion beam for 60 s repeatedly, while wide-range survey spectra and high-resolution core level spectra were taken after every etching cycle with the detector in scan mode. The ion beam was operated at 2000 V, with an approximate etching rate of 0.37 nm/s compared to Ta_2_O_5_.

Electrochemical characterization was conducted via the Ivium potentiostat. Before each electrochemical experiment run a 1.0 to 2.5 h pretreatment was conducted, where the current density was set to 400 mA/cm^2^, while the potential was monitored. After this time the potential did not change significantly anymore (ΔV < 10 mV/h), suggesting that electrochemical equilibrium has been reached. Subsequently, CP and EIS measurements were conducted from 1 to 800 mA/cm^2^ for flow cell experiments. For a precise description of the individual measurement steps, we refer to Table S2 in the SI. The data acquisition, treatment, and error determination were performed analogously to the three-electrode experiments.

### Quantification and statistical analysis

For all data with error bars, the average between the datasets was calculated using the following equation: $\frac{1}{n}=\sum_{i=i}^{n}a_{i}$.

For the same sets of data the error bars were determined via the standard deviation using the following equation: $2SD=2\cdot \sqrt{\frac{\sum {\left|x-\mu \right|}^{2}}{N}}$.
