# Supplementary material for: Effect of iron addition to the electrolyte on alkaline water electrolysis performance
Source: iScience. 2023 Dec 10;27(1):108695. doi: 10.1016/j.isci.2023.108695 (PMC10777114; doi:10.1016/j.isci.2023.108695)
Supplement: Document S1. Figures S1–S30 and Tables S1–S3 [file mmc1.pdf]

iScience, Volume *27*

Supplemental information

Effect of iron addition to the electrolyte

on alkaline water electrolysis performance

Maximilian Demnitz, Yuran Martins Lamas, Rodrigo Lira Garcia Barros, Anouk de Leeuw den Bouter, John van der Schaaf, and Matheus Theodorus de Groot

Effect of iron addition to the electrolyte on alkaline water electrolysis performance

Maximilian Demnitz[a,b*], Yuran Martins Lamas[a,b], Rodrigo Lira Garcia Barros[a,b], Anouk de Leeuw den Bouter[a,b], John van der Schaaf[a,b], Matheus Theodorus de Groot[a,b]

[a] *Department of Chemical Engineering and Chemistry, Sustainable Process Engineering Group, Eindhoven University of Technology, P.O. Box 513, Eindhoven, 5600 MB, The Netherlands*

[b] *Eindhoven Institute for Renewable Energy Systems, Eindhoven University of Technology, PO Box 513, Eindhoven 5600 MB, The Netherlands*

**Lead contact*

Maximilian Demnitz: m.demnitz@tue.nl, ORCID: 0000-0002-4137-1057

Yuran Martins Lamas: y.martins.lamas@student.tue.nl, ORCID: 0009-0000-9936-6800

Rodrigo Lira Garcia Barros: r.lira.garcia.barros@tue.nl, ORCID: 0000-0002-9227-7642

Anouk de Leeuw den Bouter: a.w.n.d.leeuw.den.bouter@tue.nl, ORCID: 0009-0003-7833-8917

John van der Schaaf: j.vanderschaaf@tue.nl, ORCID: 0000-0002-2856-8592

Matheus Theodorus de Groot: M.T.d.Groot@tue.nl, ORCID: 0000-0001-8654-8664

Keywords: Fe, industrial, temperature, KOH, hydroxide, nickel, temperature, electrolyser, HER, OER

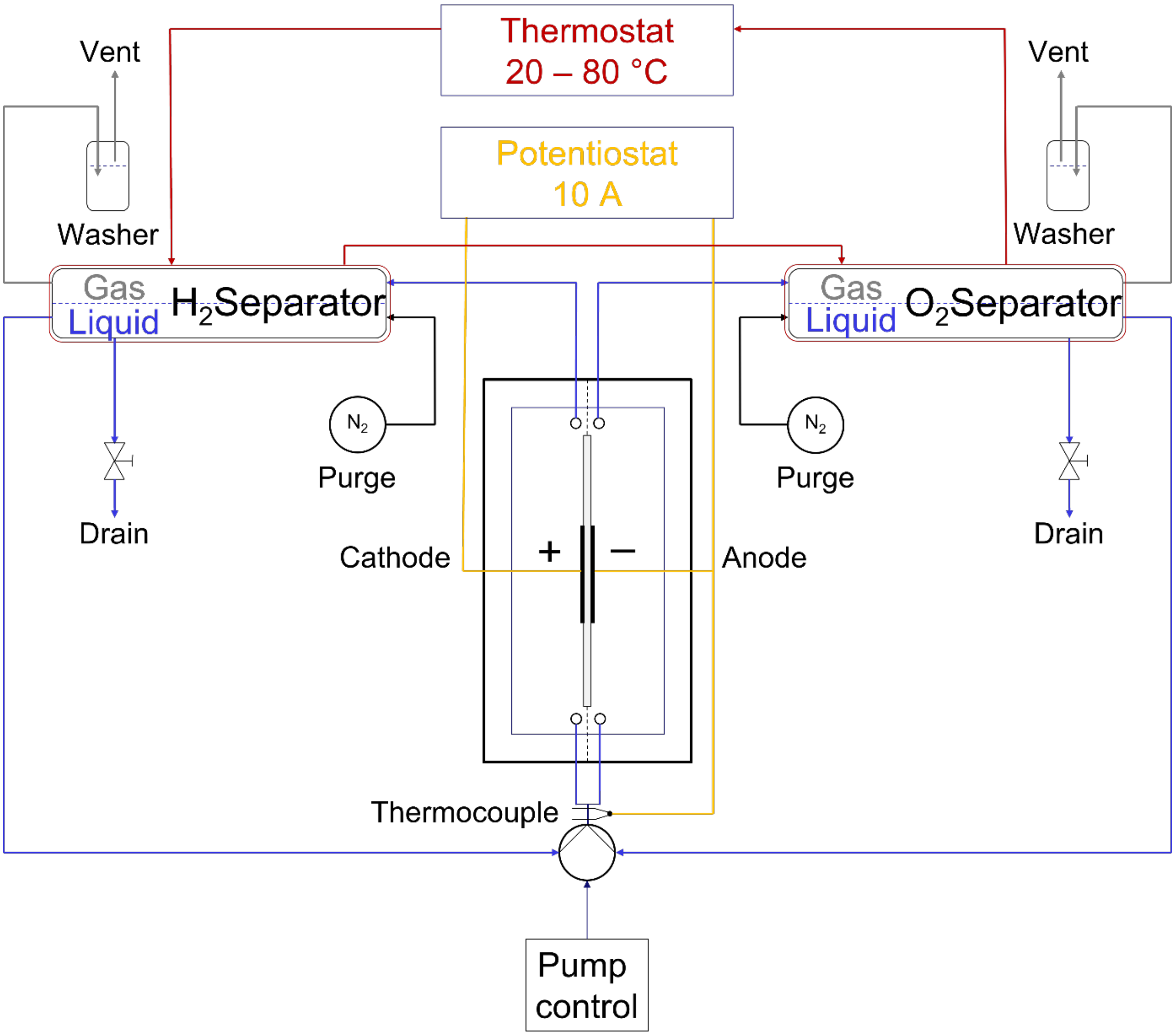

Figure S1: Schematic drawing of the flow cell setup.

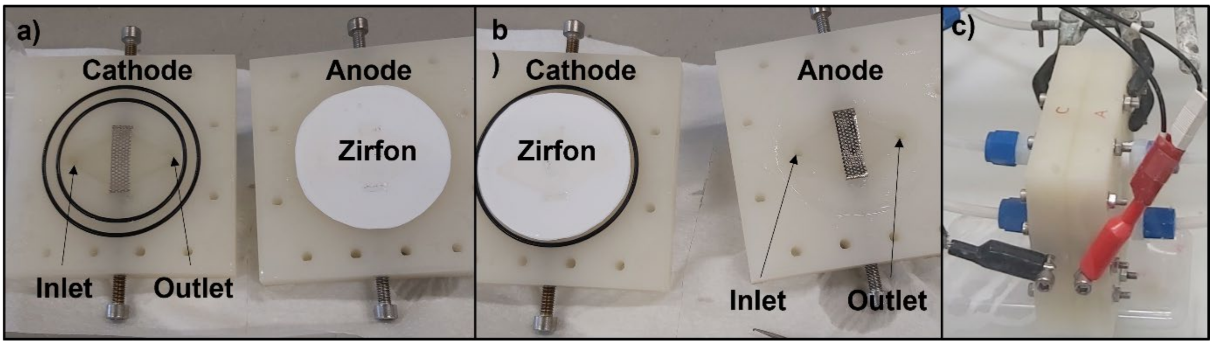

Figure S2: a) Opened flow cell with Zirfon covering the anode, b) Zirfon covering the cathode and c) assembled cell.

Table S1. Batch measurement procedure for electrochemical measurements in the 3-electrode cell. CV = Cyclic voltammetry; CP = chronopotentiometry; EIS = electrochemical impedance spectroscopy. CV for ECSA determination were conducted during the first measurement sets in the 0.2 – 0.4 V potential window and then 0.4 – 0.8 V potential window to ensure measurements under non-faradaic conditions. The loop was performed three to six times for each temperature at a fixed Fe concentration. For CP and EIS measurements the applied current was negative for HER measurements and positive for OER measurements. At each temperature (20; 50; 85 °C) the batch measurement procedure is carried out thrice consecutively to obtain triplet measurements for each individual point. The error is obtained using two times the standard deviation.

MEASUREMENT PROCEDURE

Full CV	-0.35 V to 1.60 V
Start loop	
CV	10 mV/s; 0.2 – 0.4 V
CV	20 mV/s; 0.2 – 0.4 V
CV	40 mV/s; 0.2 – 0.4 V
CV	60 mV/s; 0.2 – 0.4 V
CV	80 mV/s; 0.2 – 0.4 V
CV	100 mV/s; 0.2 – 0.4 V
CV	120 mV/s; 0.2 – 0.4 V
CV	140 mV/s; 0.2 – 0.4 V
CV	160 mV/s; 0.2 – 0.4 V
CV	180 mV/s; 0.2 – 0.4 V
CV	200 mV/s; 0.2 – 0.4 V
CV	250 mV/s; 0.2 – 0.4 V
CV	300 mV/s; 0.2 – 0.4 V
CV	450 mV/s; 0.2 – 0.4 V
CV	400 mV/s; 0.2 – 0.4 V
Pretreatment CP	400 mA/cm^2 for 1 h
CP	800 mA/cm^2 for 60 s
EIS	800 mA/cm^2
CP	600 mA/cm^2 for 60 s
EIS	600 mA/cm^2
CP	400 mA/cm^2 for 60 s
EIS	400 mA/cm^2
CP	200 mA/cm^2 for 60 s
EIS	200 mA/cm^2
CP	100 mA/cm^2 for 60 s
EIS	100 mA/cm^2
CP	80 mA/cm^2 for 60 s
EIS	80 mA/cm^2
CP	60 mA/cm^2 for 60 s
EIS	60 mA/cm^2
CP	40 mA/cm^2 for 60 s
EIS	40 mA/cm^2
CP	20 mA/cm^2 for 60 s
EIS	20 mA/cm^2
CP	10 mA/cm^2 for 60 s
EIS	10 mA/cm^2
CP	8 mA/cm^2 for 60 s
EIS	8 mA/cm^2

CP	6 mA/cm^2 for 60 s
EIS	6 mA/cm^2
CP	4 mA/cm^2 for 60 s
EIS	4 mA/cm^2
CP	2 mA/cm^2 for 60 s
EIS	2 mA/cm^2
CP	1 mA/cm^2 for 60 s
EIS	1 mA/cm^2
CP	0.5 mA/cm^2 for 60 s
EIS	0.5 mA/cm^2
CV	10 mV/s; 0.2 – 0.4 V
CV	20 mV/s; 0.2 – 0.4 V
CV	40 mV/s; 0.2 – 0.4 V
CV	60 mV/s; 0.2 – 0.4 V
CV	80 mV/s; 0.2 – 0.4 V
CV	100 mV/s; 0.2 – 0.4 V
CV	120 mV/s; 0.2 – 0.4 V
CV	140 mV/s; 0.2 – 0.4 V
CV	160 mV/s; 0.2 – 0.4 V
CV	180 mV/s; 0.2 – 0.4 V
CV	200 mV/s; 0.2 – 0.4 V
CV	250 mV/s; 0.2 – 0.4 V
CV	300 mV/s; 0.2 – 0.4 V
CV	450 mV/s; 0.2 – 0.4 V
CV	400 mV/s; 0.2 – 0.4 V
End loop	
Full CV	-0.35 V to 1.60 V

Table S2. Batch measurement procedure for electrochemical measurements in the flow cell. CP = chronopotentiometry; EIS = electrochemical impedance spectroscopy. At each temperature (20; 50; 85 °C) the batch measurement procedure is carried out thrice consecutively to obtain triplet measurements for each individual point. The error is obtained using two times the standard deviation.

**MEASUREMENT PROCEDURE**	
Pretreatment CP	400 mA/cm^2 for 1 h
CP	800 mA/cm^2 for 60 s
EIS	800 mA/cm^2
CP	600 mA/cm^2 for 60 s
EIS	600 mA/cm^2
CP	400 mA/cm^2 for 60 s
EIS	400 mA/cm^2
CP	200 mA/cm^2 for 60 s
EIS	200 mA/cm^2
CP	100 mA/cm^2 for 60 s
EIS	100 mA/cm^2
CP	80 mA/cm^2 for 60 s
EIS	80 mA/cm^2
CP	60 mA/cm^2 for 60 s
EIS	60 mA/cm^2
CP	40 mA/cm^2 for 60 s
EIS	40 mA/cm^2
CP	20 mA/cm^2 for 60 s
EIS	20 mA/cm^2
CP	10 mA/cm^2 for 60 s
EIS	10 mA/cm^2
CP	8 mA/cm^2 for 60 s
EIS	8 mA/cm^2
CP	6 mA/cm^2 for 60 s
EIS	6 mA/cm^2
CP	4 mA/cm^2 for 60 s
EIS	4 mA/cm^2
CP	2 mA/cm^2 for 60 s
EIS	2 mA/cm^2
CP	1 mA/cm^2 for 60 s
EIS	1 mA/cm^2
CP	0.5 mA/cm^2 for 60 s
EIS	0.5 mA/cm^2

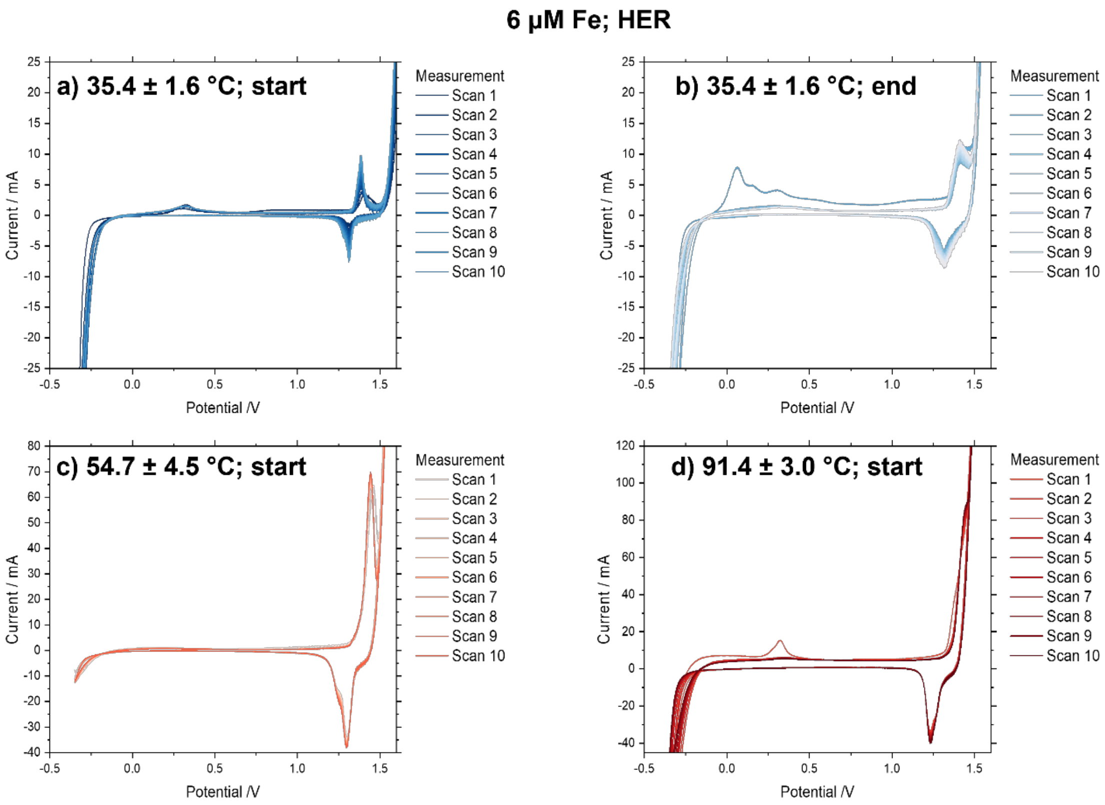

Figure S3. Full cyclic voltammograms for HER obtained for 30 wt.% KOH at 6 µM Fe obtained at a) the start of the 35 °C experimental batch procedure, b) the end of the 35 °C experimental batch procedure, c) the start of the 55 °C experimental batch procedure, and d) the start of the 91 °C experimental batch procedure. The end measurements at 55 and 91 °C were unfortunately not measured due to an error with the potentiostat software.

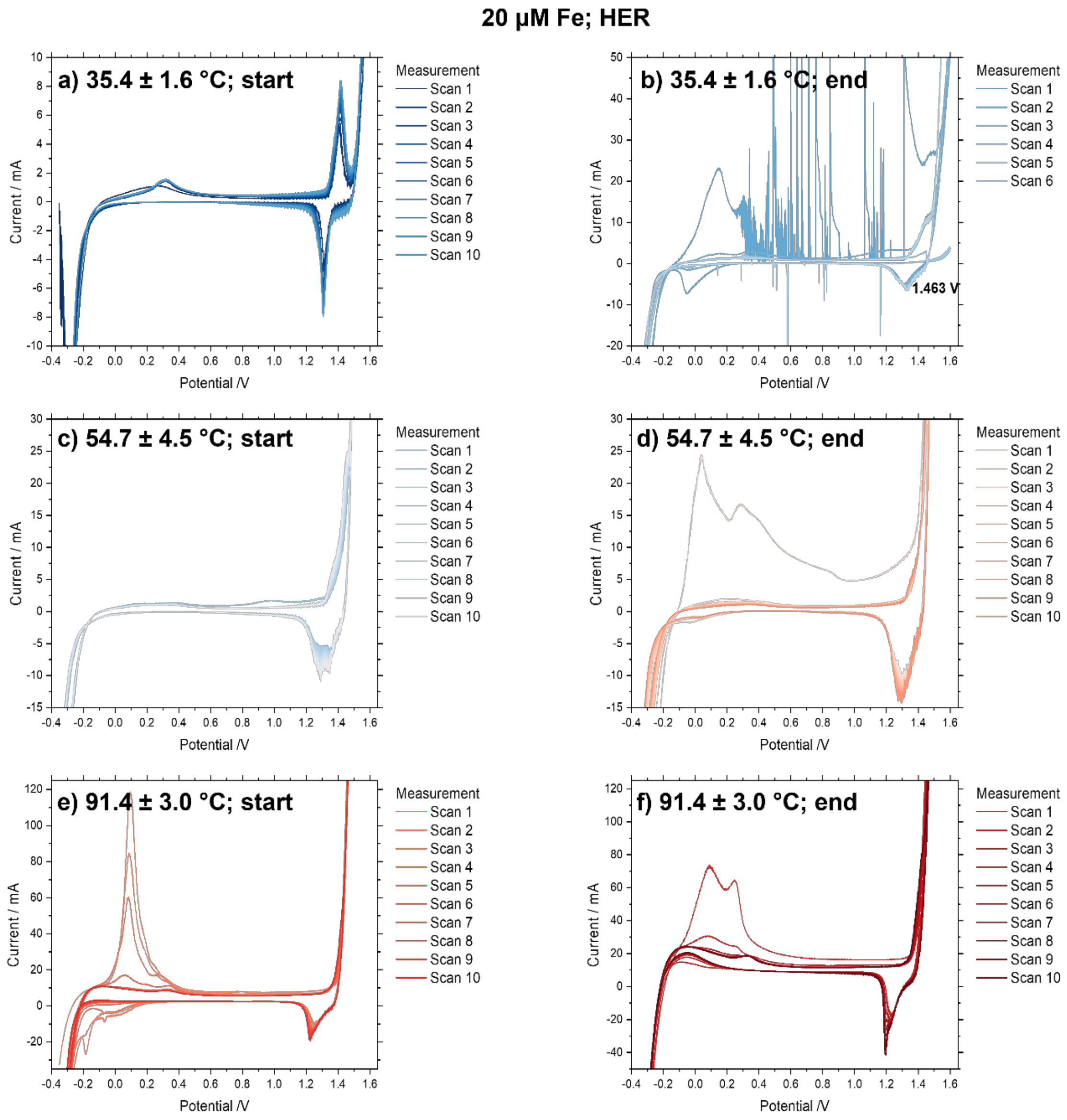

Figure S4. Full cyclic voltammograms for HER obtained for 30 wt.% KOH at 20 µM Fe obtained at a) the start of the 35 °C experimental batch procedure, b) the end of the 35 °C experimental batch procedure, c) the start of the 55 °C experimental batch procedure, d) the end of the 55 °C experimental batch procedure, e) the start of the 91 °C experimental batch procedure, f) the end of the 91 °C experimental batch procedure.

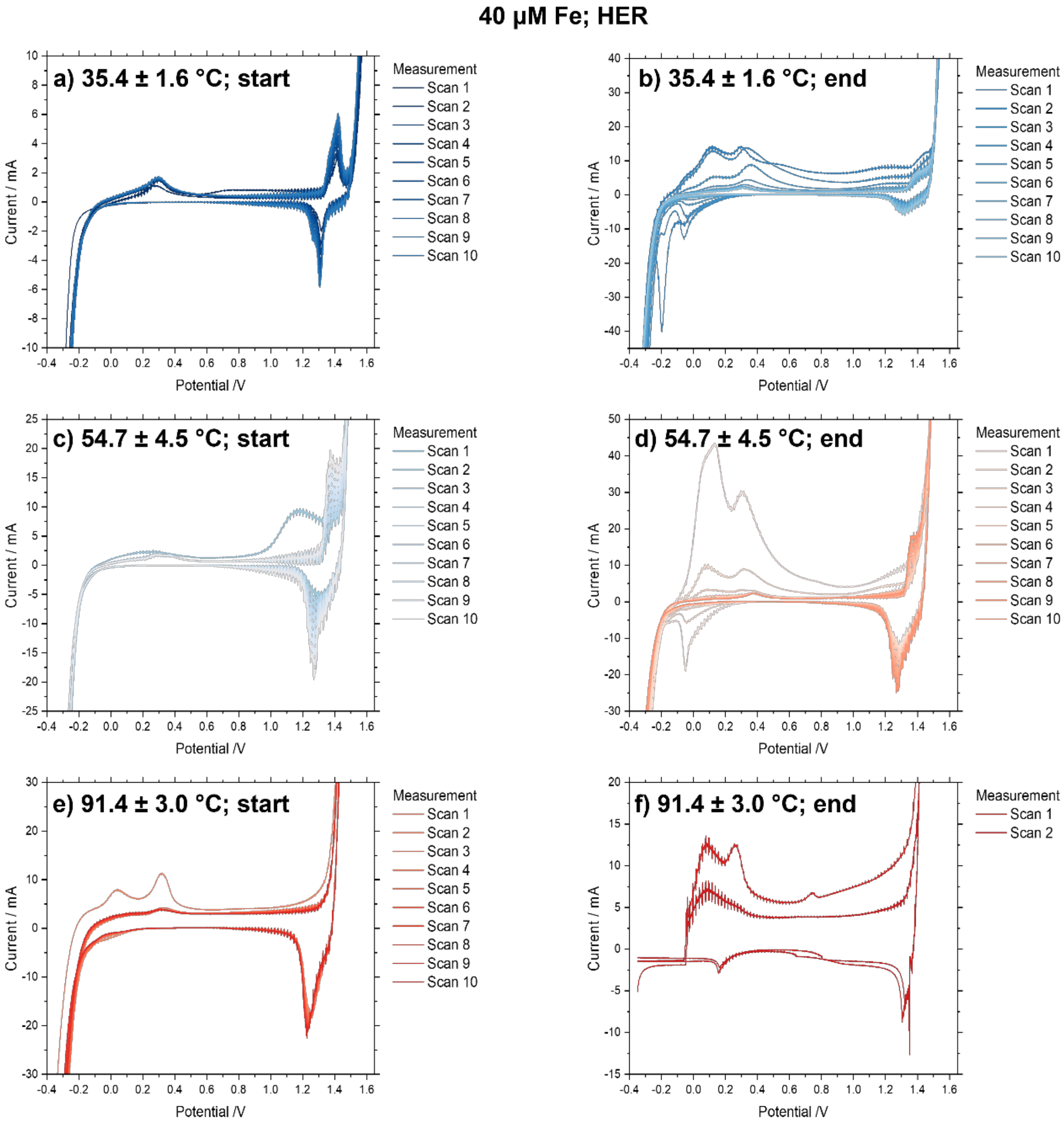

Figure S5. Full cyclic voltammograms for HER obtained for 30 wt.% KOH at 40 µM Fe obtained at a) the start of the 35 °C experimental batch procedure, b) the end of the 35 °C experimental batch procedure, c) the start of the 55 °C experimental batch procedure, d) the end of the 55 °C experimental batch procedure, e) the start of the 91 °C experimental batch procedure, f) the end of the 91 °C experimental batch procedure.

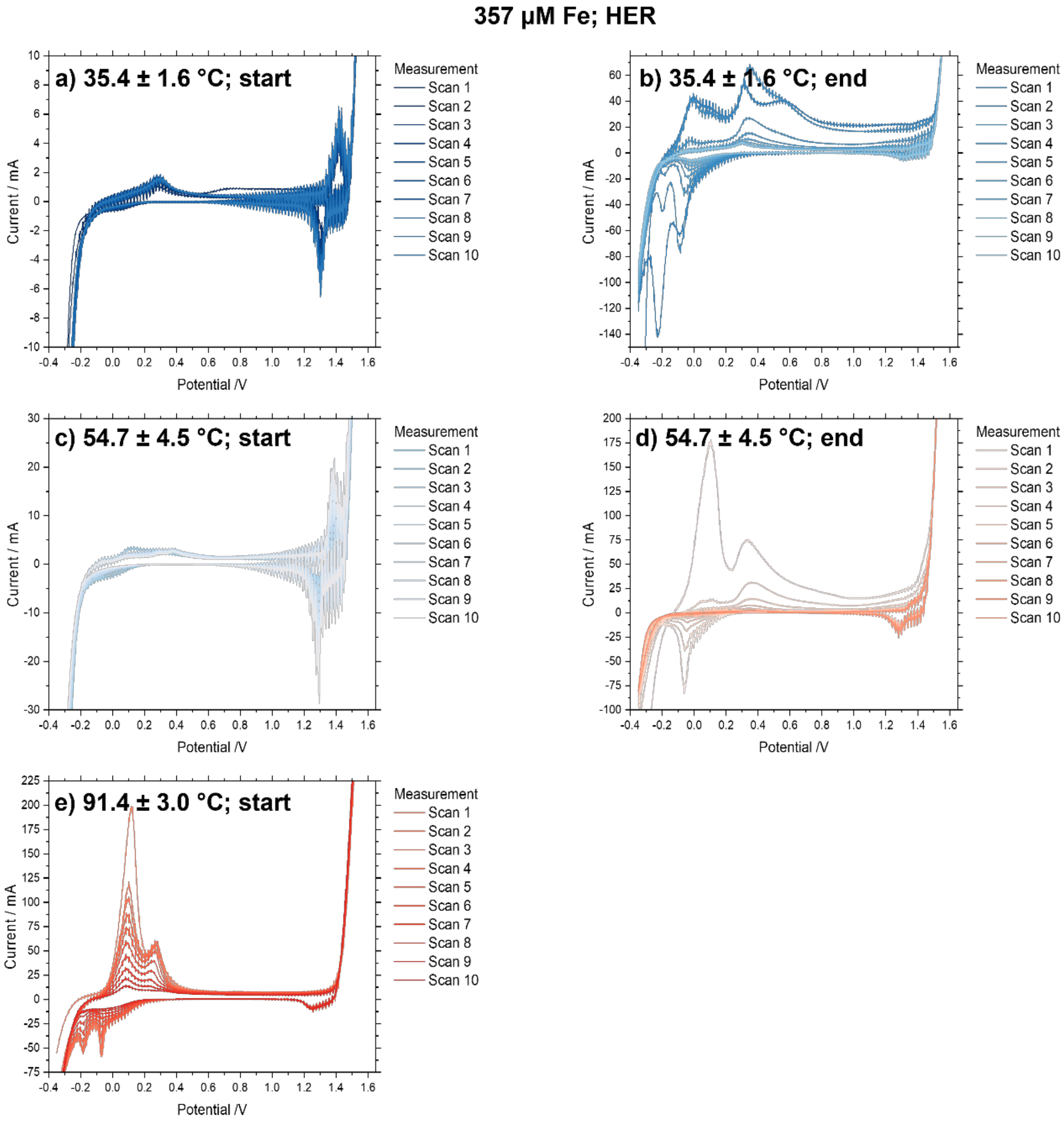

Figure S6. Full cyclic voltammograms for HER obtained for 30 wt.% KOH at 357 µM Fe obtained at a) the start of the 35 °C experimental batch procedure, b) the end of the 35 °C experimental batch procedure, c) the start of the 55 °C experimental batch procedure, d) the end of the 55 °C experimental batch procedure, and e) the start of the 91a °C experimental batch procedure. The end measurements at 91 °C were unfortunately not measured due to an error with the potentiostat software.

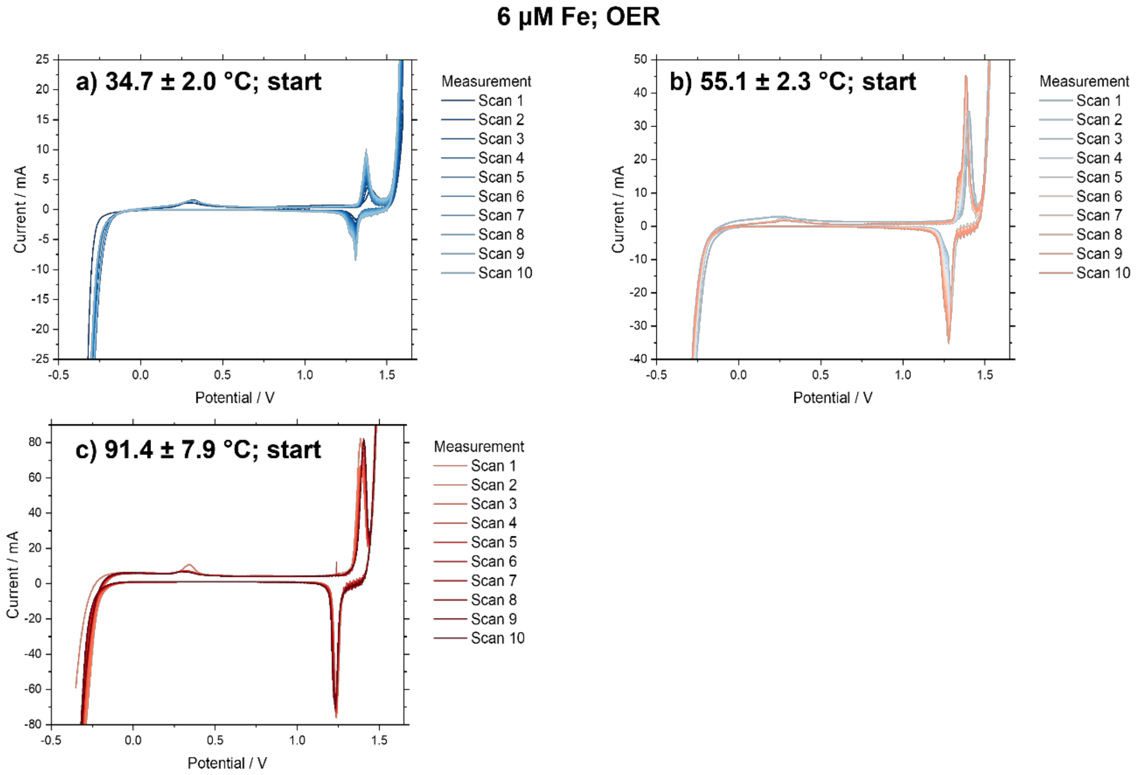

Figure S7. Full cyclic voltammograms for OER obtained for 30 wt.% KOH at 6 µM Fe obtained at a) the start of the 35 °C experimental batch procedure, b) the start of the 55 °C experimental batch procedure, and c) the start of the 91 °C experimental batch procedure. The end measurements at 34, 55 and 91 °C were unfortunately not measured due to an error with the potentiostat software.

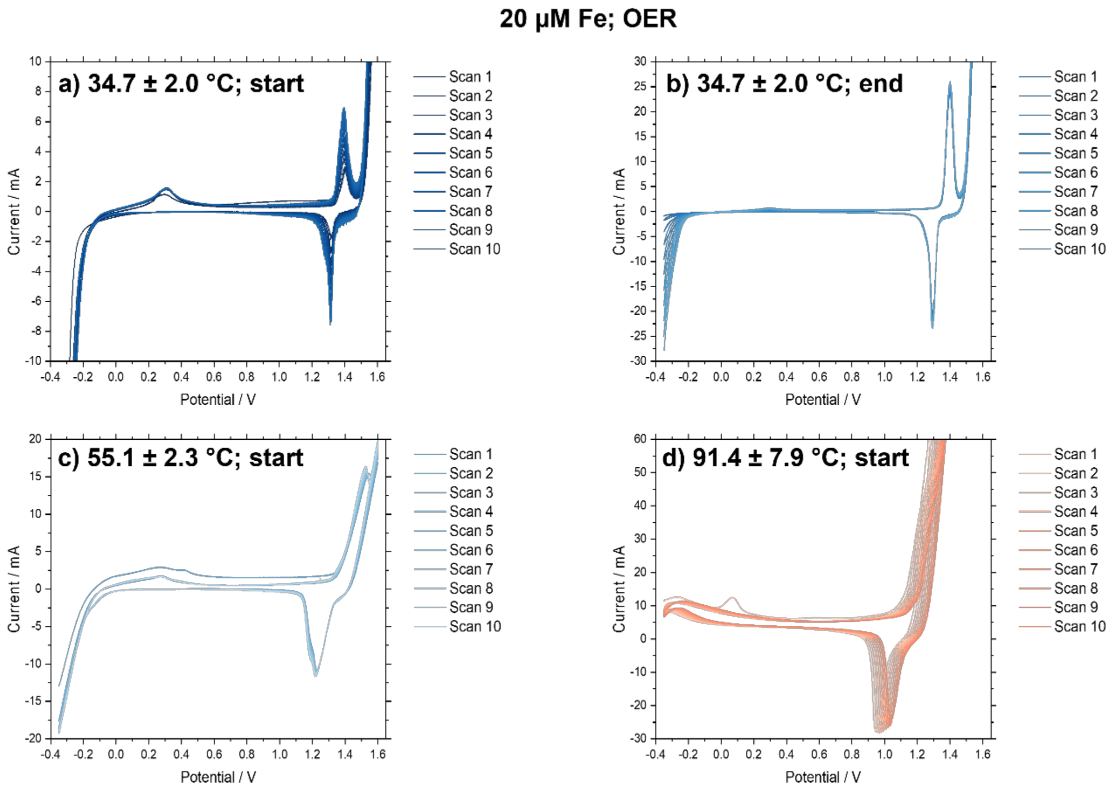

Figure S8. Full cyclic voltammograms for OER obtained for 30 wt.% KOH at 20 µM Fe obtained at a) the start of the 35 °C experimental batch procedure, b) the end of the 35 °C experimental batch procedure, c) the start of the 55 °C experimental batch procedure, and d) the start of the 91 °C experimental batch procedure. The end measurements at 55 and 91 °C were unfortunately not measured due to an error with the potentiostat software.

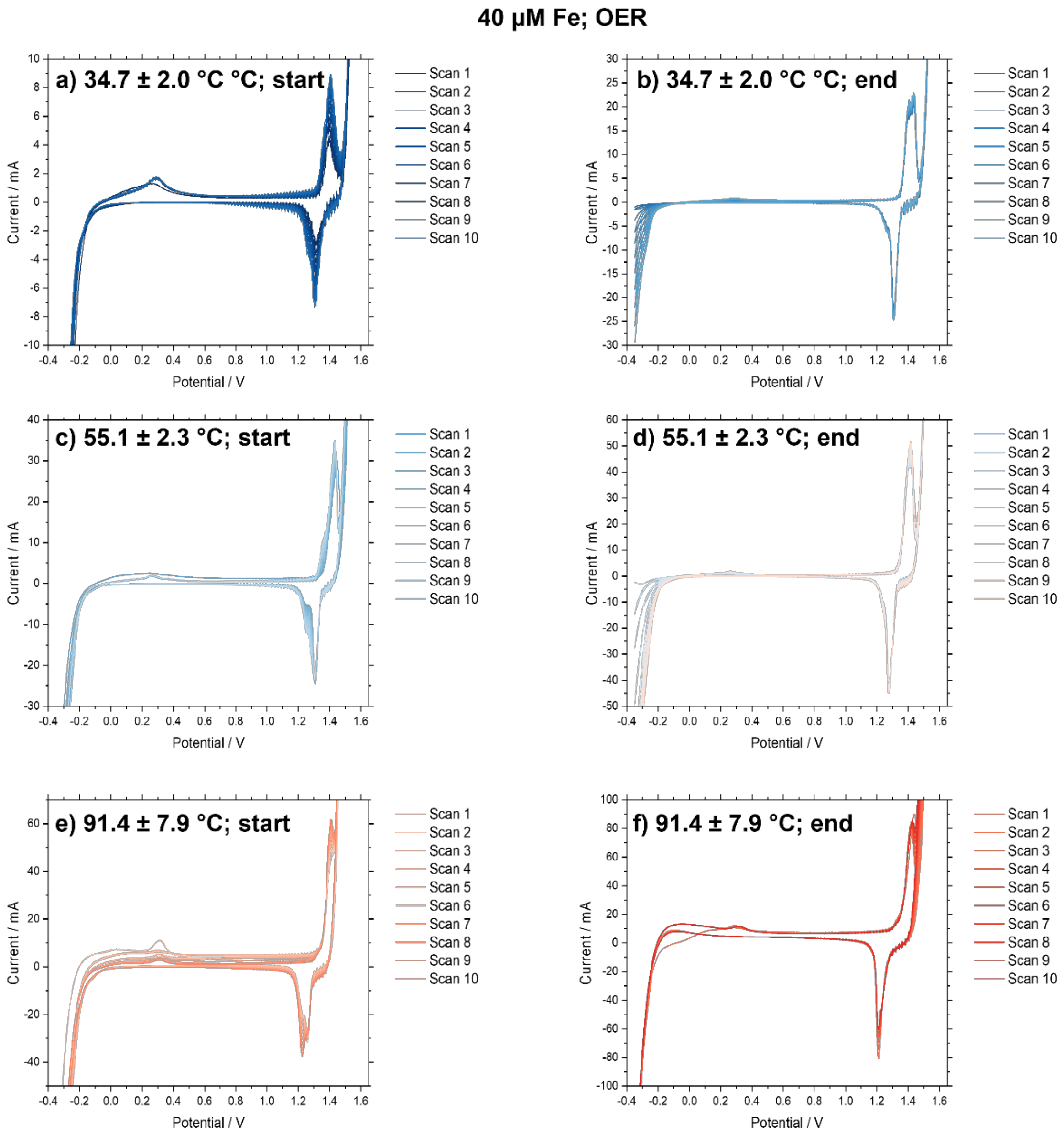

Figure S9. Full cyclic voltammograms for OER obtained for 30 wt.% KOH at 40 µM Fe obtained at a) the start of the 35 °C experimental batch procedure, b) the end of the 35 °C experimental batch procedure, c) the start of the 55 °C experimental batch procedure, d) the end of the 55 °C experimental batch procedure, e) the start of the 91 °C experimental batch procedure, f) the end of the 91 °C experimental batch procedure.

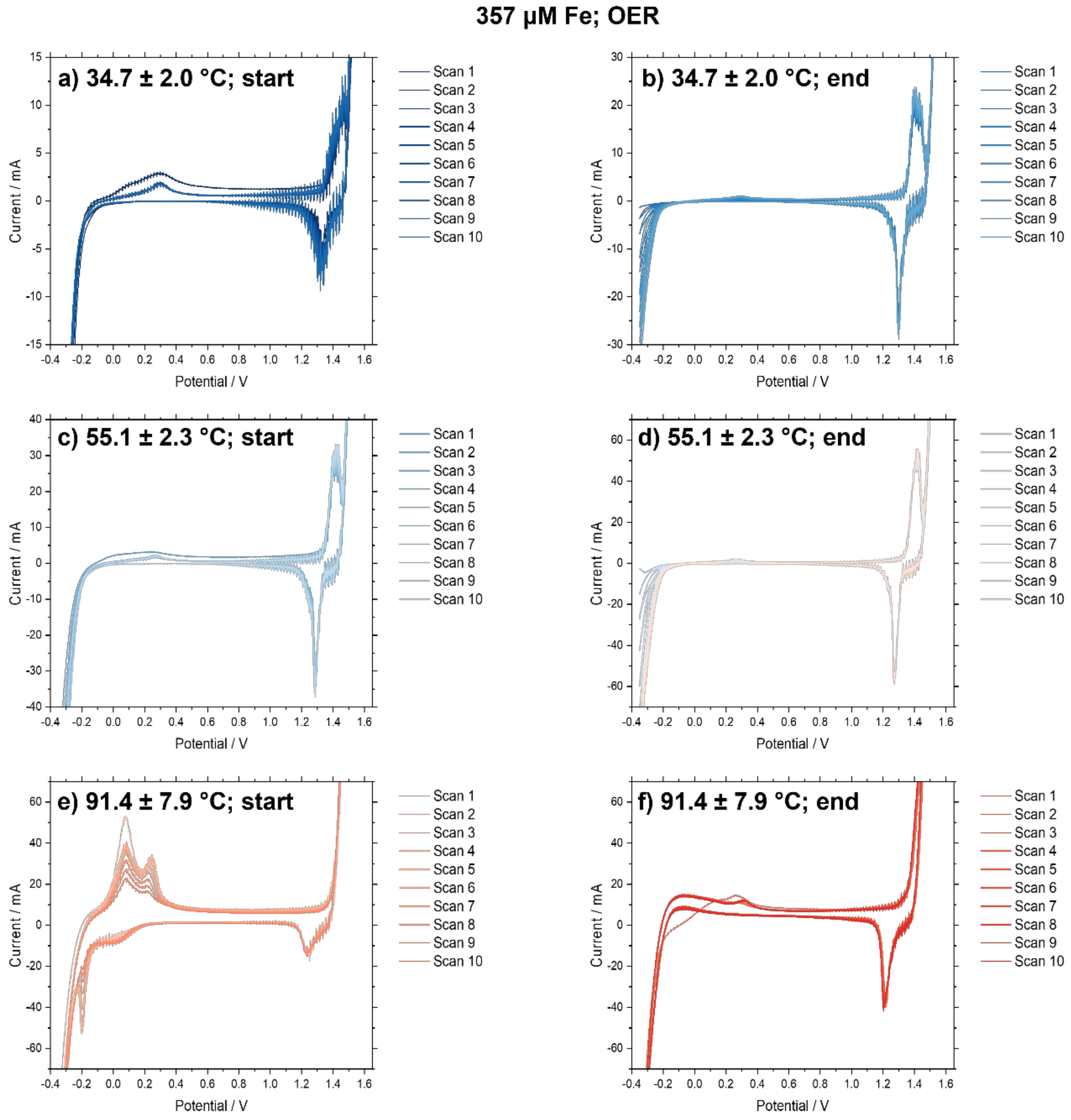

Figure S10. Full cyclic voltammograms for OER obtained for 30 wt.% KOH at 357 µM Fe obtained at a) the start of the 35 °C experimental batch procedure, b) the end of the 35 °C experimental batch procedure, c) the start of the 55 °C experimental batch procedure, d) the end of the 55 °C experimental batch procedure, e) the start of the 91 °C experimental batch procedure, f) the end of the 91 °C experimental batch procedure.

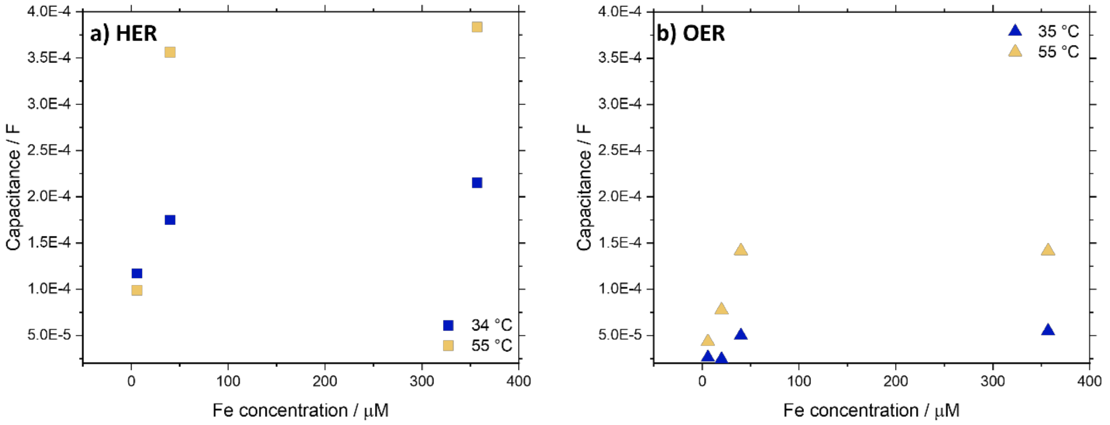

Figure S11. Capacitance determined in the non-faradaic region using cyclic voltammetry at scan speeds between 10 – 400 mV/s for working electrodes used after a) HER and b) OER conditioning at Fe concentrations of 6 – 357 µM and T = 35 – 55 °C for a 2 cm^2 electrode.

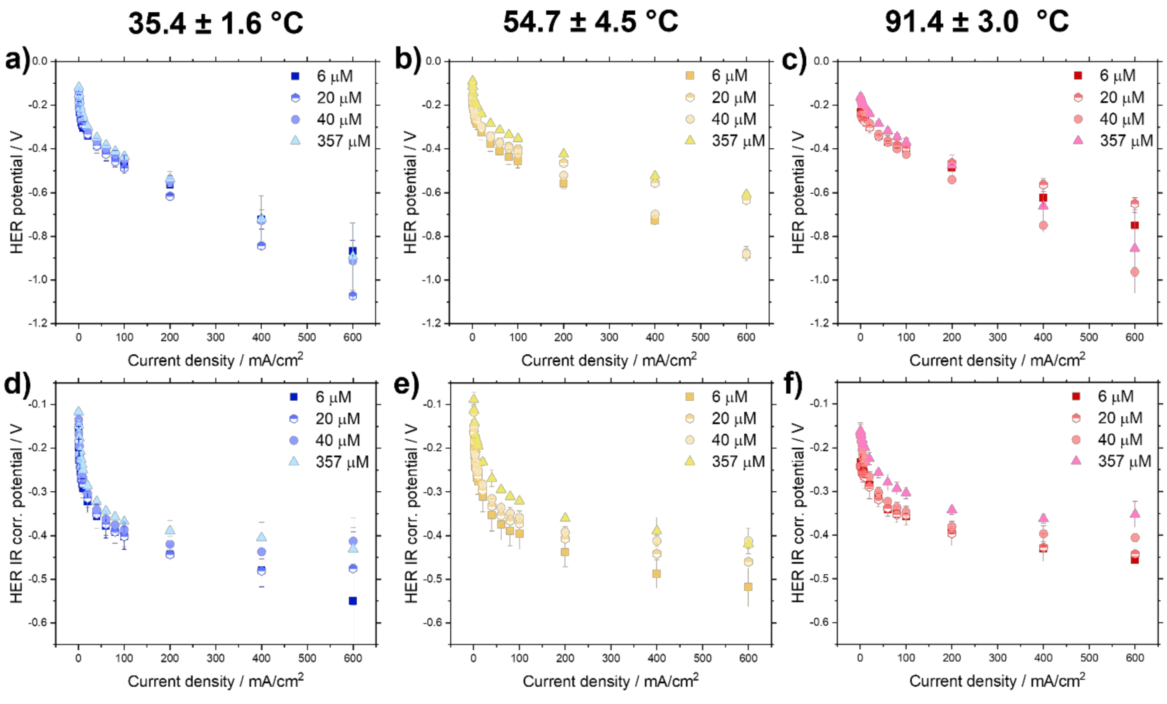

Figure S12. IV curves determined from chronopotentiometry in the 3-electrode cell for HER with 30 wt.% KOH with Fe conc. of 6, 20, 40, and 357 µM at a) 35, b) 55, and c) 91 °C as well as IV curves with the internal resistance corrected potentials for the same electrolyte compositions at d) 35, e) 55, and f) 91 °C. Data are represented as mean ± 2 standard deviations.

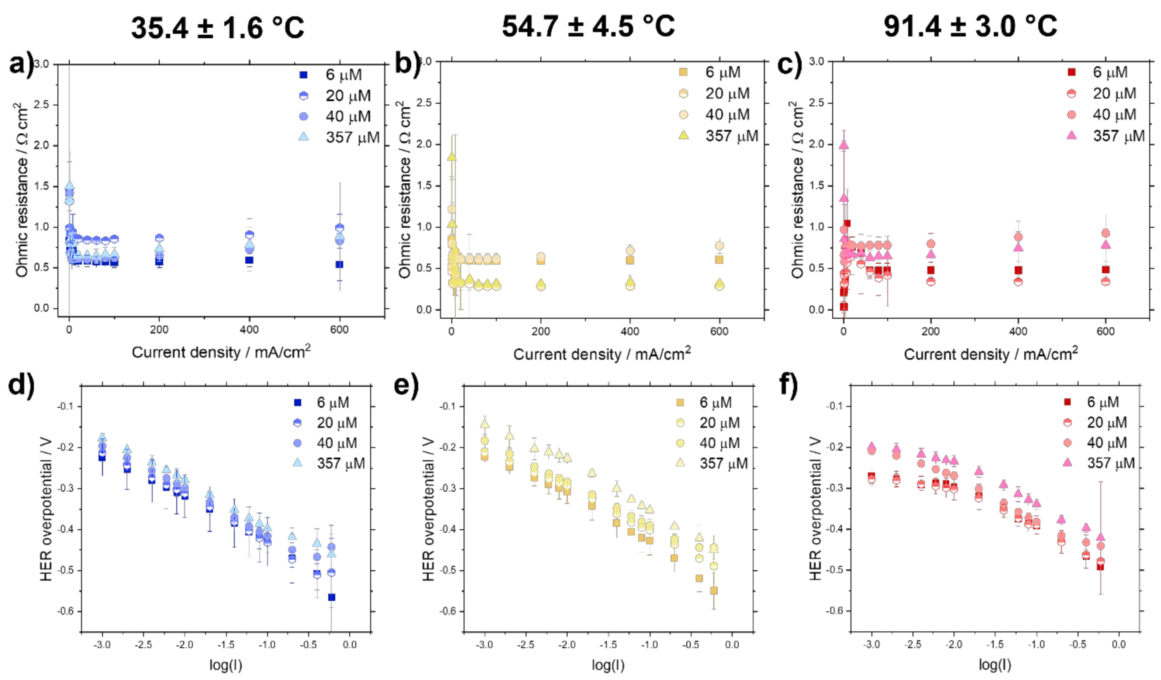

Figure S13. Ohmic resistances determined from EIS in the 3-electrode cell for HER with 30 wt.% KOH with Fe conc. of 6, 20, 40, and 357 μM at a) 35, b) 55, and c) 91 °C as well as Tafel slopes for the same electrolyte compositions at d) 35, e) 55, and f) 91 °C. Note that for the Tafel plots the logarithmic value of the current density was used in A, not mA. Data are represented as mean ± 2 standard deviations.

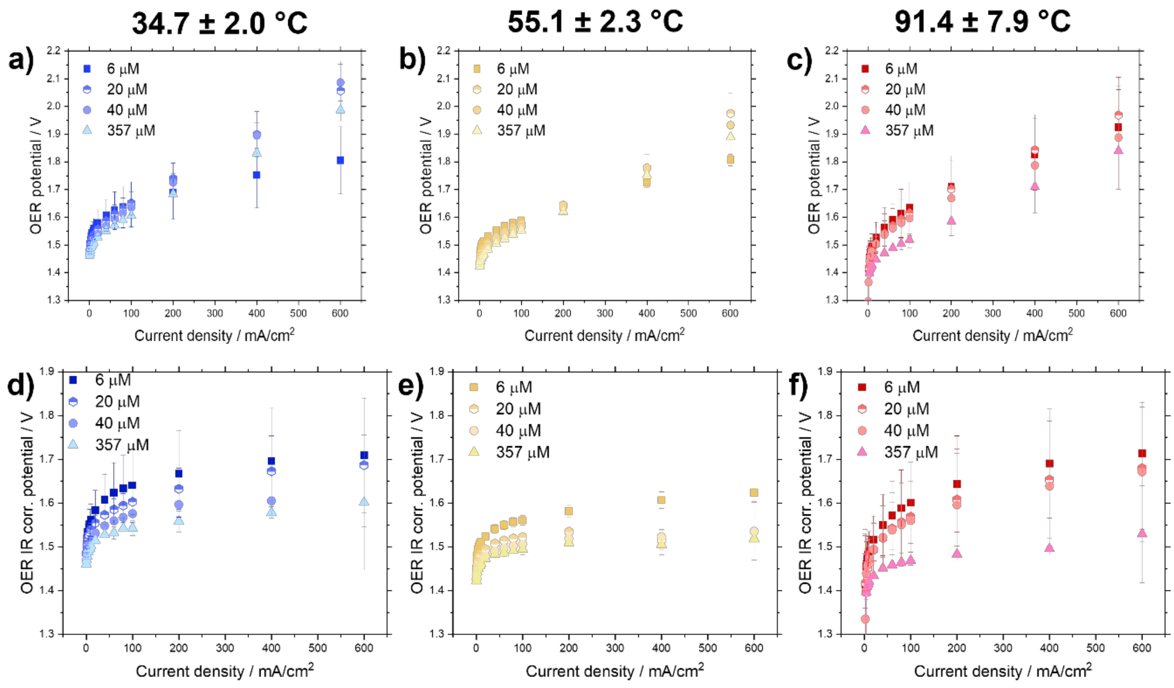

Figure S14. IV curves determined from chronopotentiometry in the 3-electrode cell for OER with 30 wt.% KOH with Fe conc. of 6, 20, 40, and 357 μM at a) 35, b) 55, and c) 91 °C as well as IV curves with the internal resistance corrected potentials for the same electrolyte compositions at d) 35, e) 55, and f) 91 °C. Data are represented as mean ± 2 standard deviations.

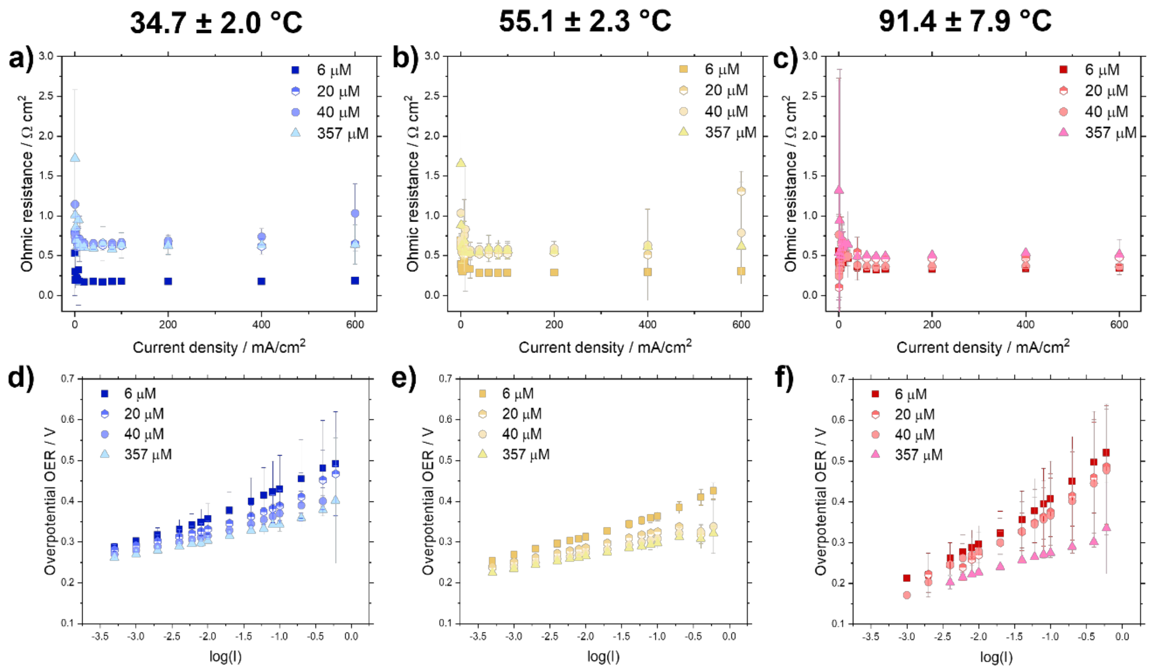

Figure S15. Ohmic resistances determined from EIS in the 3-electrode cell for OER with 30 wt.% KOH with Fe conc. of 6, 20, 40, and 357 µM at a) 35, b) 55, and c) 91°C as well as Tafel slopes for the same electrolyte compositions at d) 35, e) 55, and f) 91 °C. Note that for the Tafel plots the logarithmic value of the current density was used in A, not mA. Data are represented as mean ± 2 standard deviations.

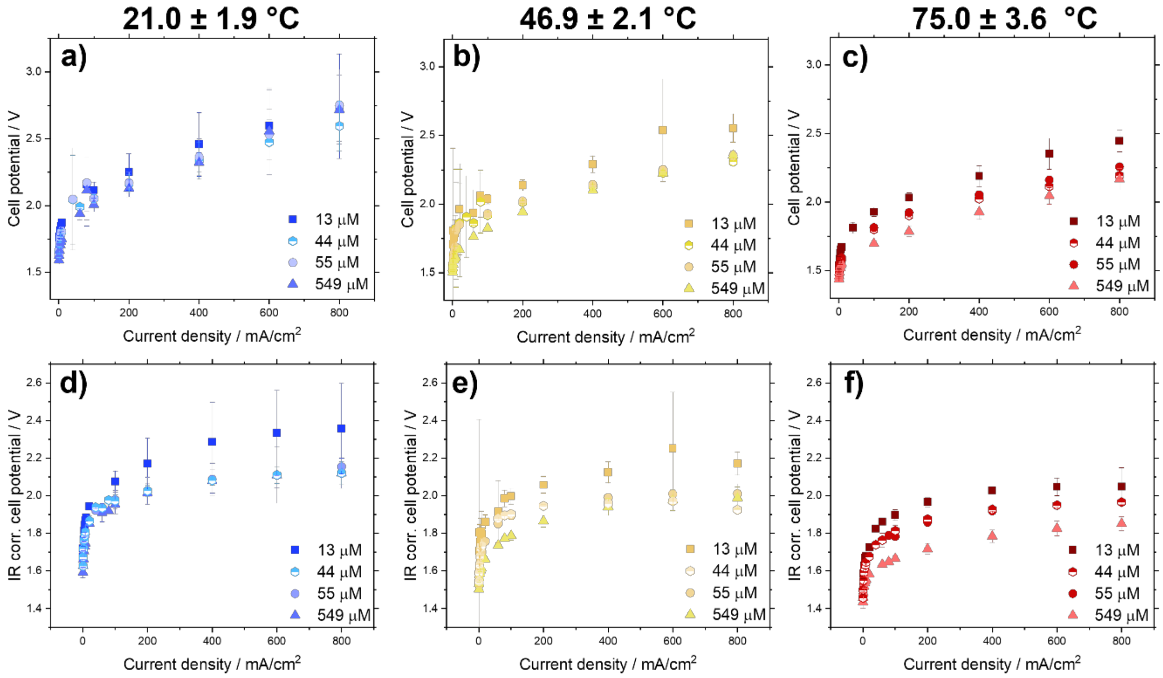

Figure S16. IV curves determined from chronopotentiometry in a flow cell with 30 wt.% KOH with Fe conc. of 13, 44, 55, and 549 µM at a) 21.0 ± 1.9, b) 46.9 ± 2.1, and c) 75.0 ± 3.6 °C as well as IV curves with the internal resistance corrected potentials for the same electrolyte compositions at d) 21.0 ± 1.9, e) 46.9 ± 2.1, and f) 75.0 ± 3.6 °C. Data are represented as mean ± 2 standard deviations.

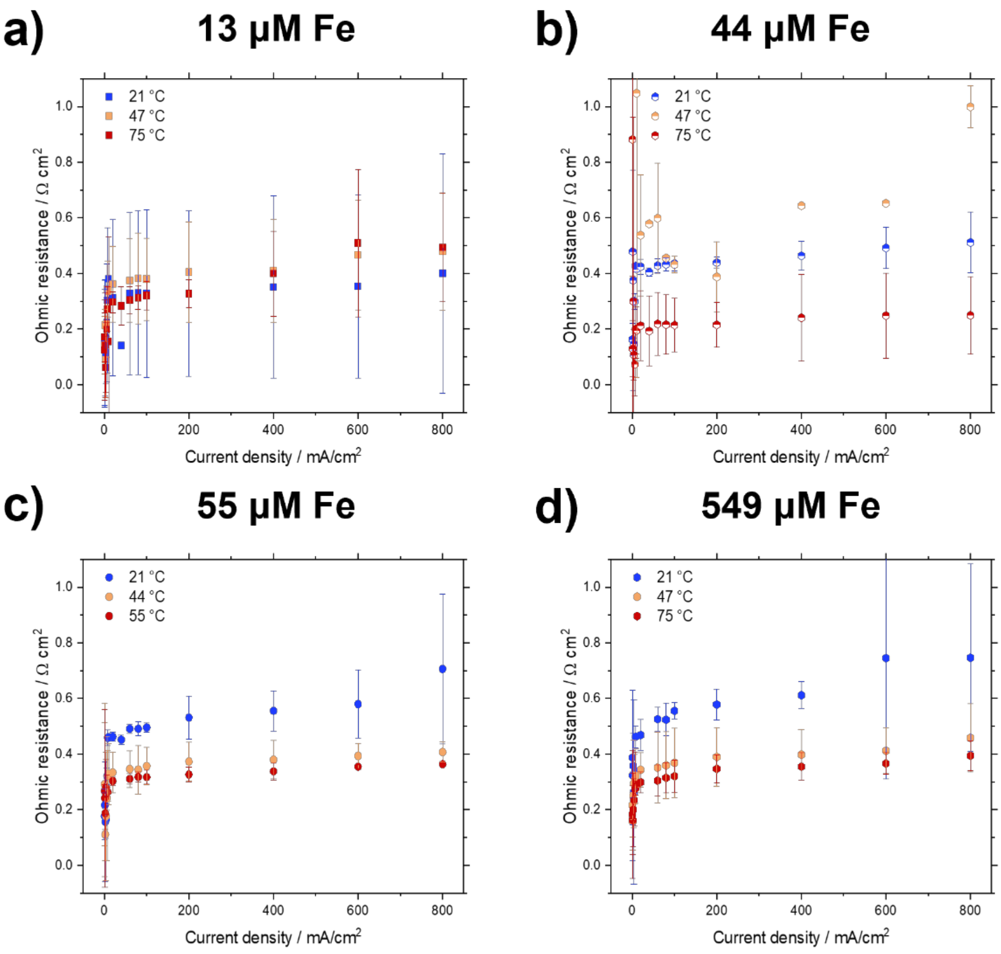

Figure S17. Ohmic resistances for different temperatures for the flow cell with 30 wt.% KOH with an added Fe concentration of a) 13 µM, b) 44 µM, c) 55 µM, and d) 549 µM. Data are represented as mean ± 2 standard deviations.

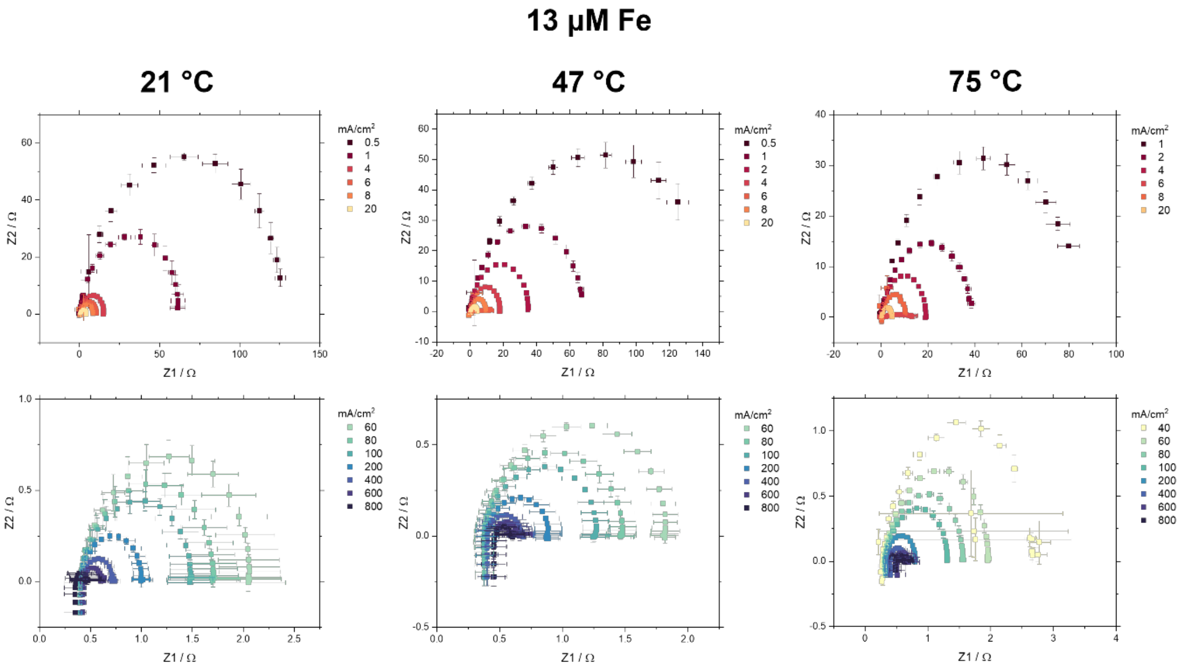

119

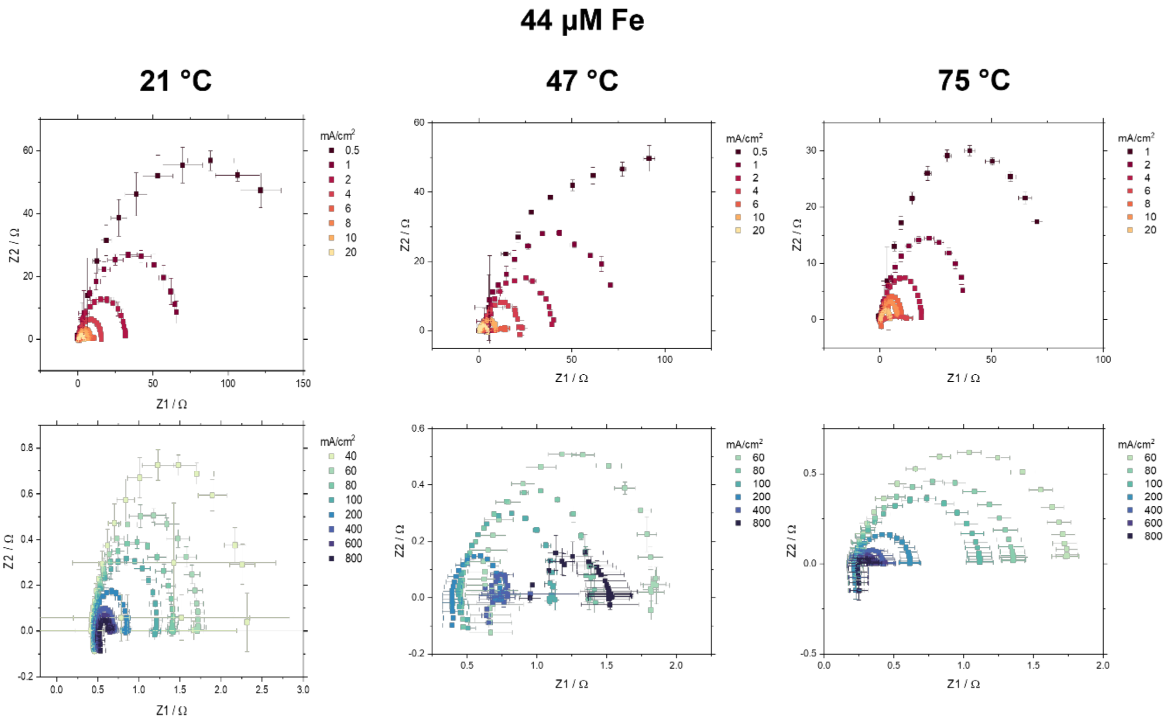
44 µM Fe
21 °C
47 °C
75 °C
mA/cm²
Z1 / Ω
Z2 / Ω

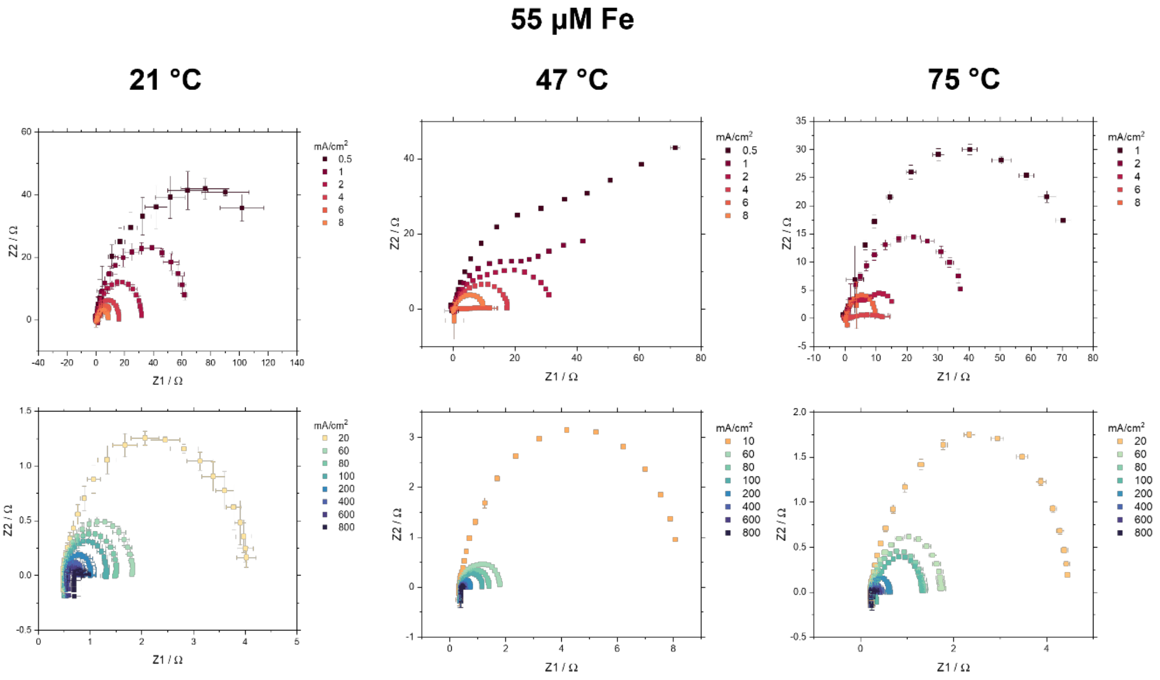
55 µM Fe
21 °C
47 °C
75 °C
mA/cm²
Z1 / Ω
Z2 / Ω

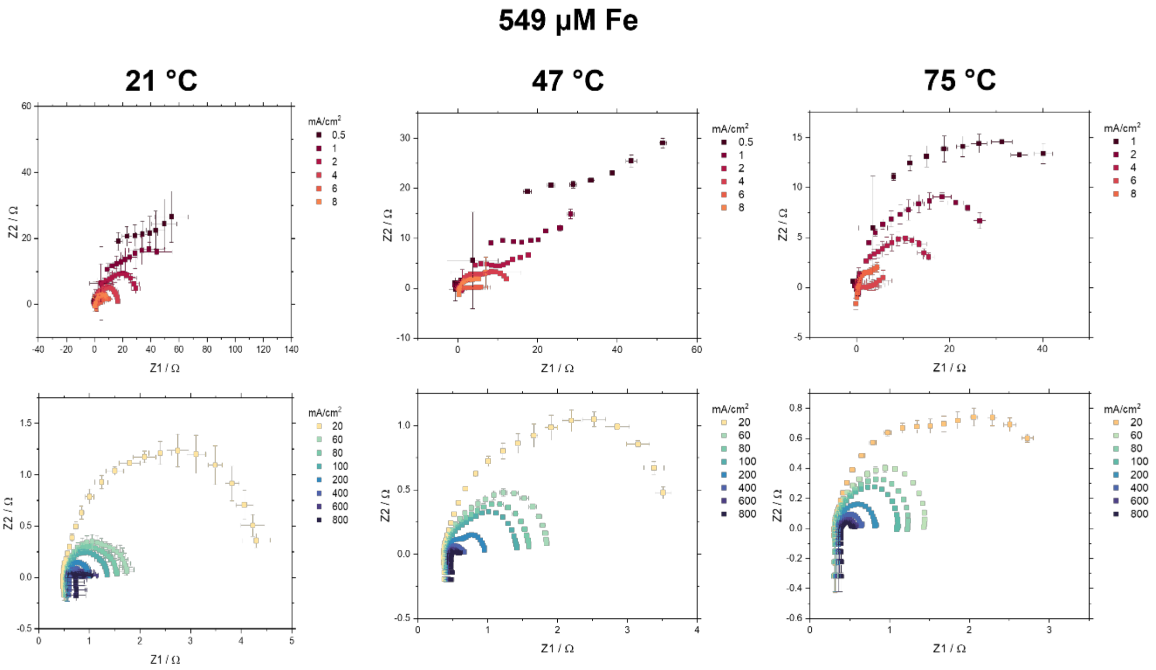

Figure S18. EIS spectra obtained at current densities between 0.5 – 800 mA/cm2 for different temperatures for the flow cell with 30 wt.% KOH with an added Fe concentration of 13 µM, 44 µM, 55 µM, and 549 µM The EIS spectra for each current density were are the averages of three runs with the error bars showing the 2σ Z1 and Z2. Singular runs were omitted due to potentiostat failure at certain current density ranges. Data are represented as mean ± 2 standard deviations.

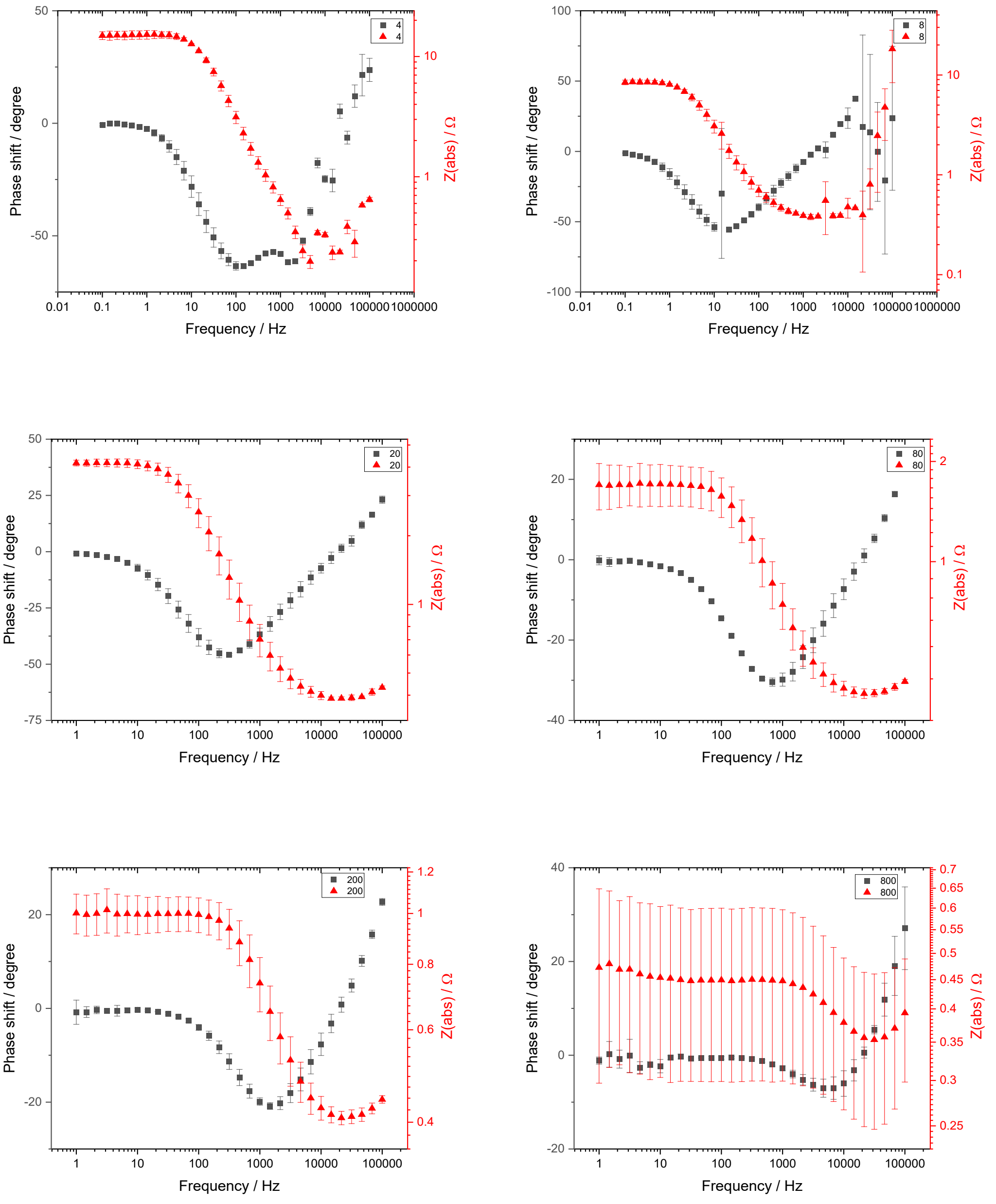

Figure S19. Exemplary Bode plots obtained at 13 μM Fe concentration at 21.0 ± 1.9 °C at 4, 8, 20, 80, 200, and 800 mA/cm^2 using EIS. Data are represented as mean ± 2 standard deviations.

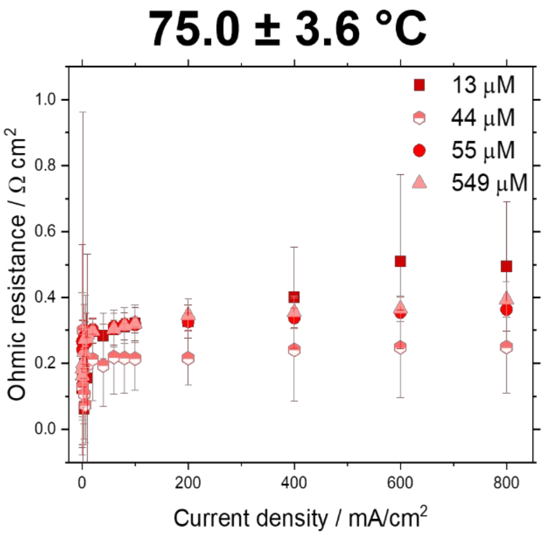

132

133 Figure 20. Ohmic resistances at 75.0 ± 3.6 °C for the flow cell with 30 wt.% KOH with Fe concentrations of 13, 44, 55, and
134 549 μM. Please note that experiments at 13, 55, and 549 μM Fe concentration were conducted with an older iteration of the
135 flow cell, while experiments at 44 μM Fe concentration were conducted with a new iteration of the flow cell. Data are
136 represented as mean ± 2 standard deviations.

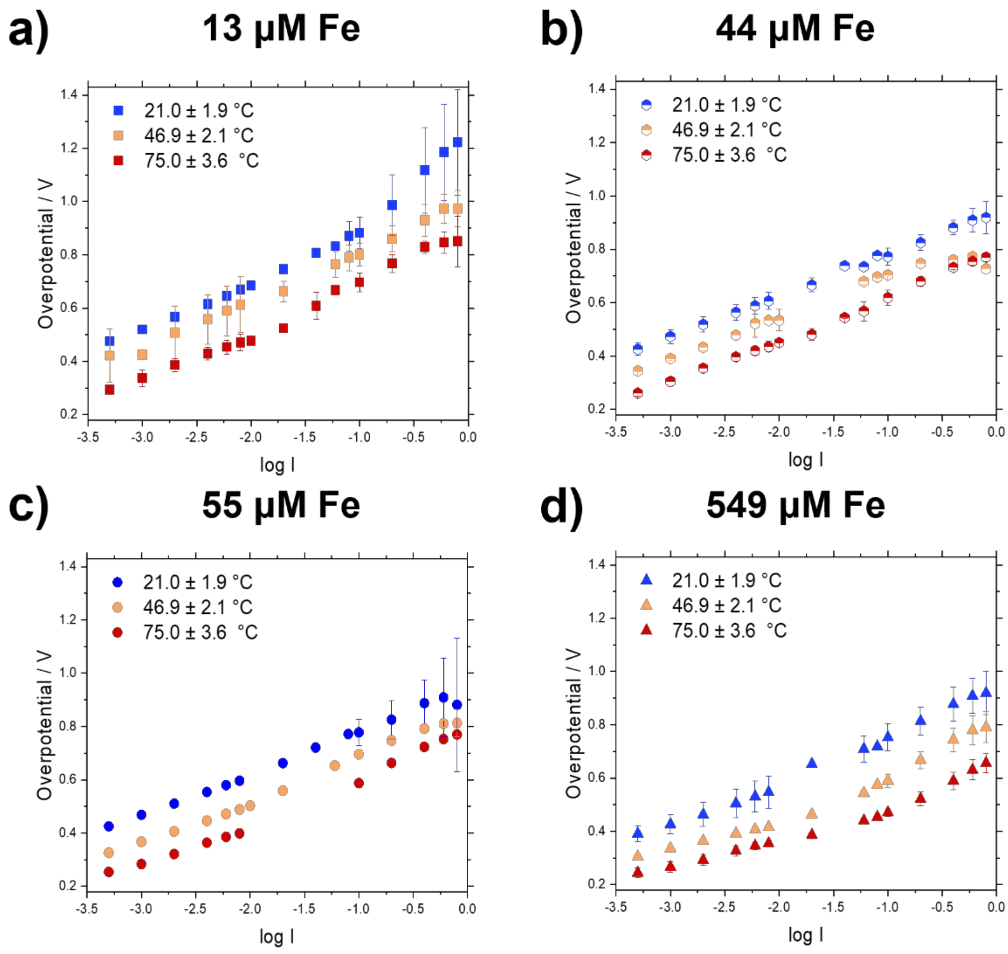

Figure S21. Tafel slopes at 21.0 ± 1.9, 46.9 ± 2.1, and 75.0 ± 3.6 °C for the flow cell with 30 wt.% KOH with an Fe concentration of a) 13, b) 44, c) 55, and d) 549 µM. Note that for the Tafel plots the logarithmic value of the current density was used in A, not mA. Data are represented as mean ± 2 standard deviations.

Table S3. Values and formulas used to calculate the limiting current density for Fe reduction on the cathode.

Density KOH 30%	1.2813	g/cm^3	1281300	g/m^3	1281.3	kg/m^3
Dynamic viscosity KOH 30%	2.357	mPa s	0.002357	Pa s	kg/m s	
Flow electrolyte	5.44	mL/s	cm^3/s			
Area electrode	2.5	cm^2				
Velocity of electrolyte flow (v)	2.176	cm/s	0.02176	m/s		
Width electrode (B)	30	mm	0.03	m		
Thickness electrode (S)	10	mm	0.01	m		
d_e electrode	0.015	m				

Using $d_e = l = 2 \cdot \frac{BS}{(B+S)}$

v (KOH 30%) =	1.83954E-06	m^2/s
D (Fe(II)) =	7.19E-10	m^2/s

Sc =	2558.5
Re =	177.4

$$S_C = \frac{\mathrm{v}}{\mathrm{D}}$$

$$R_e = \frac{\mathrm{vl}}{\nu}$$

Sh =	74.9

$$S_h = \frac{k_L l}{D}$$

n=	2	e^-				
F=	96485.33	C/mol				
c=	0.000549	mol/L	0.549	mol/m^3		
**I=**	**3.80E-01**	**A/m^2**	**380**	**mA/m^2**	**0.038**	**mA/cm^2**

$$I = nFc\frac{S_h D}{l}$$

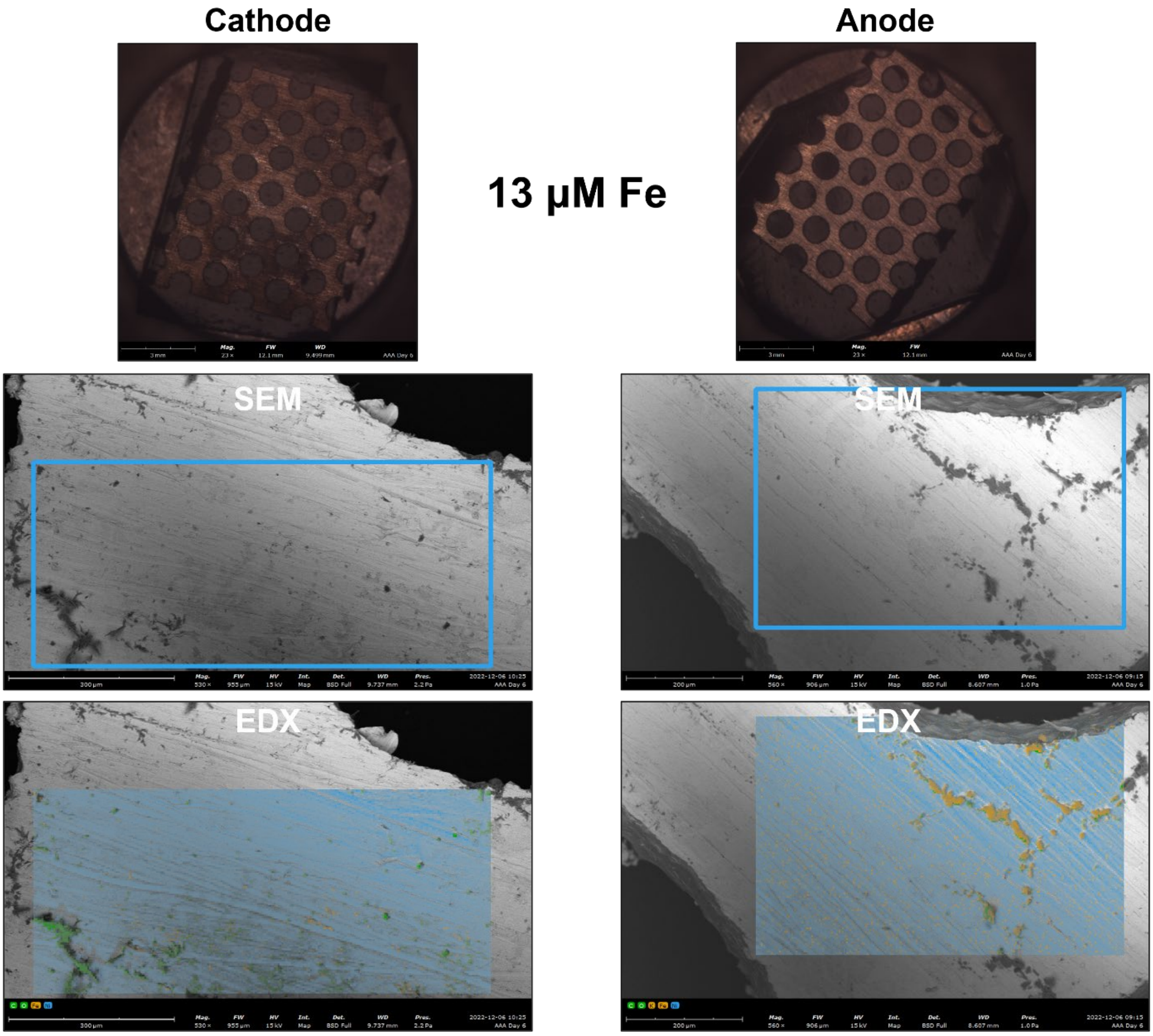

Figure S22. Optical, SEM and EDX images of the cathode and anode post electrolysis using 13 µM Fe in the electrolyte.

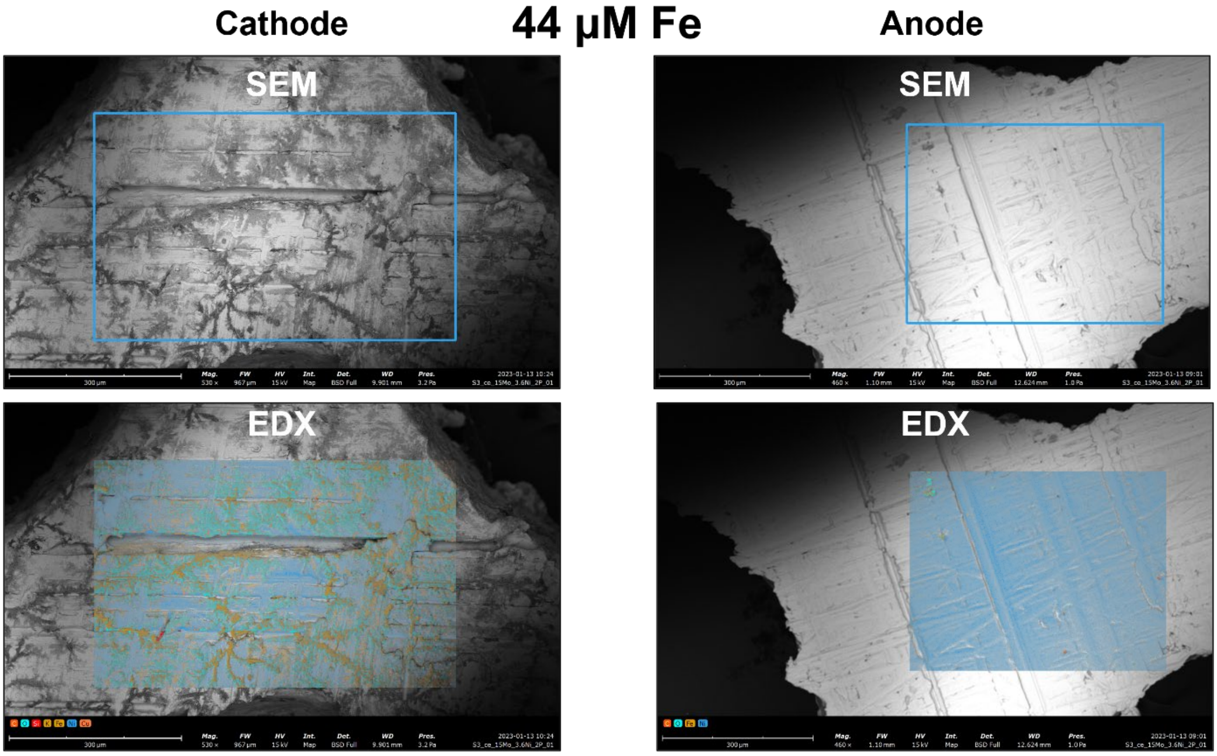

Figure S23. SEM and EDX images of the cathode and anode post electrolysis using 44 µM Fe in the electrolyte.

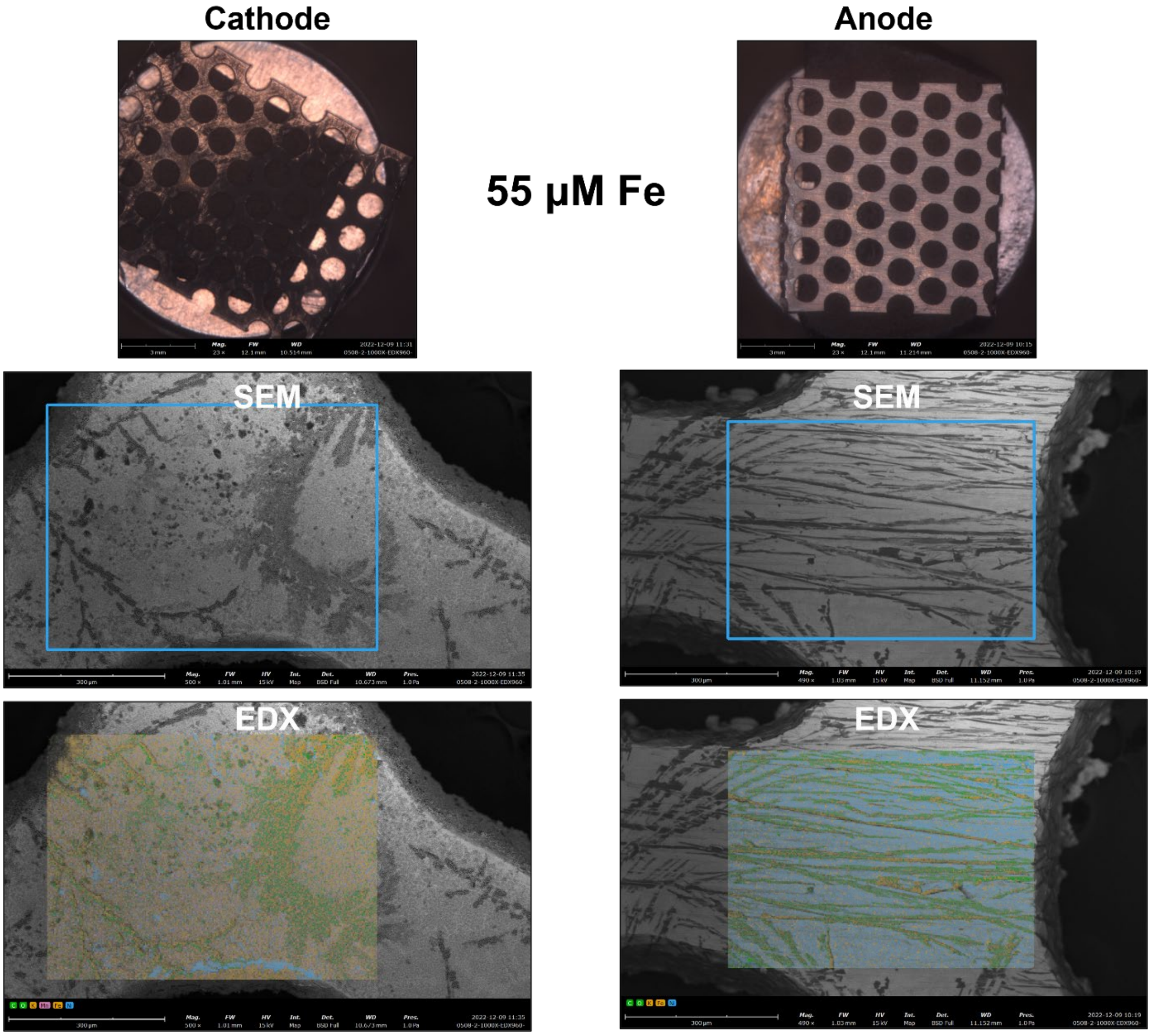

Figure S24. Optical, SEM and EDX images of the cathode and anode post electrolysis using 55 µM Fe in the electrolyte.

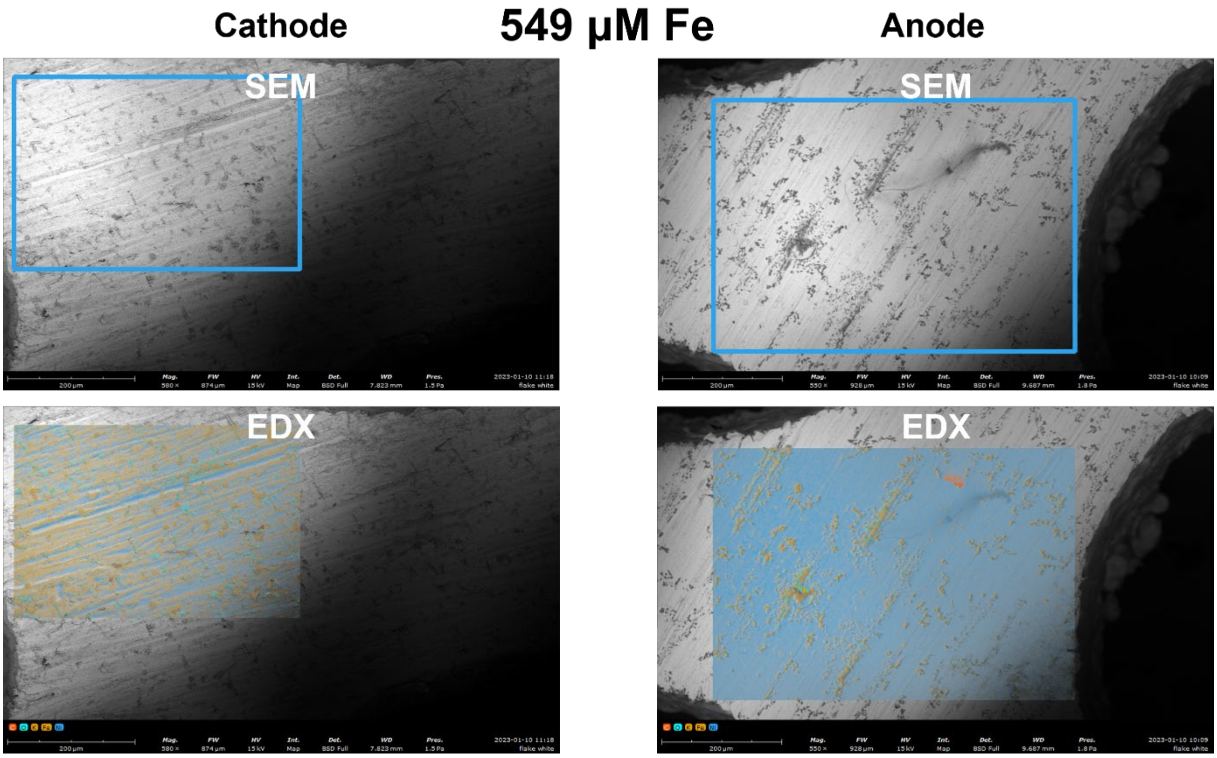

Figure S25. SEM and EDX images of the cathode and anode post electrolysis using 549 µM Fe in the electrolyte.

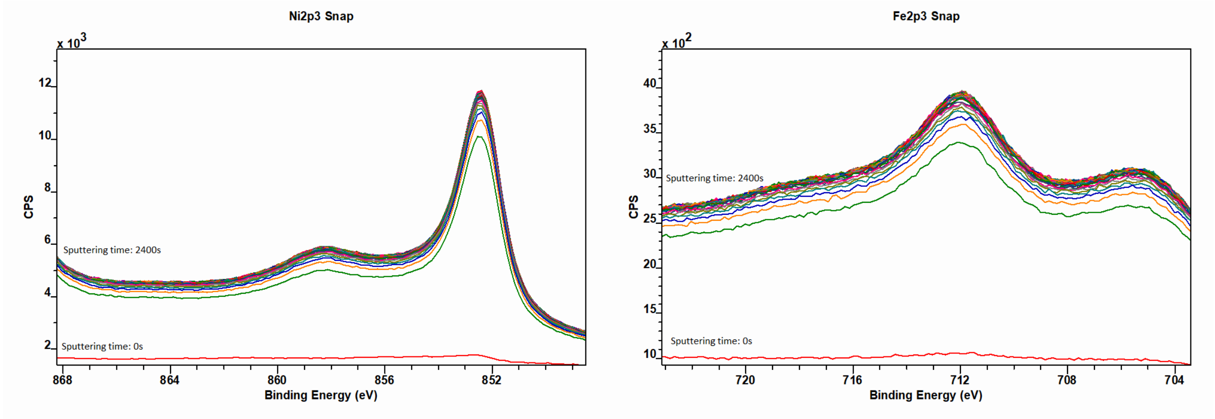

Figure S26. XPS spectra of the Ni-2p[3] and Fe-2p[3] peaks of the pristine electrode.

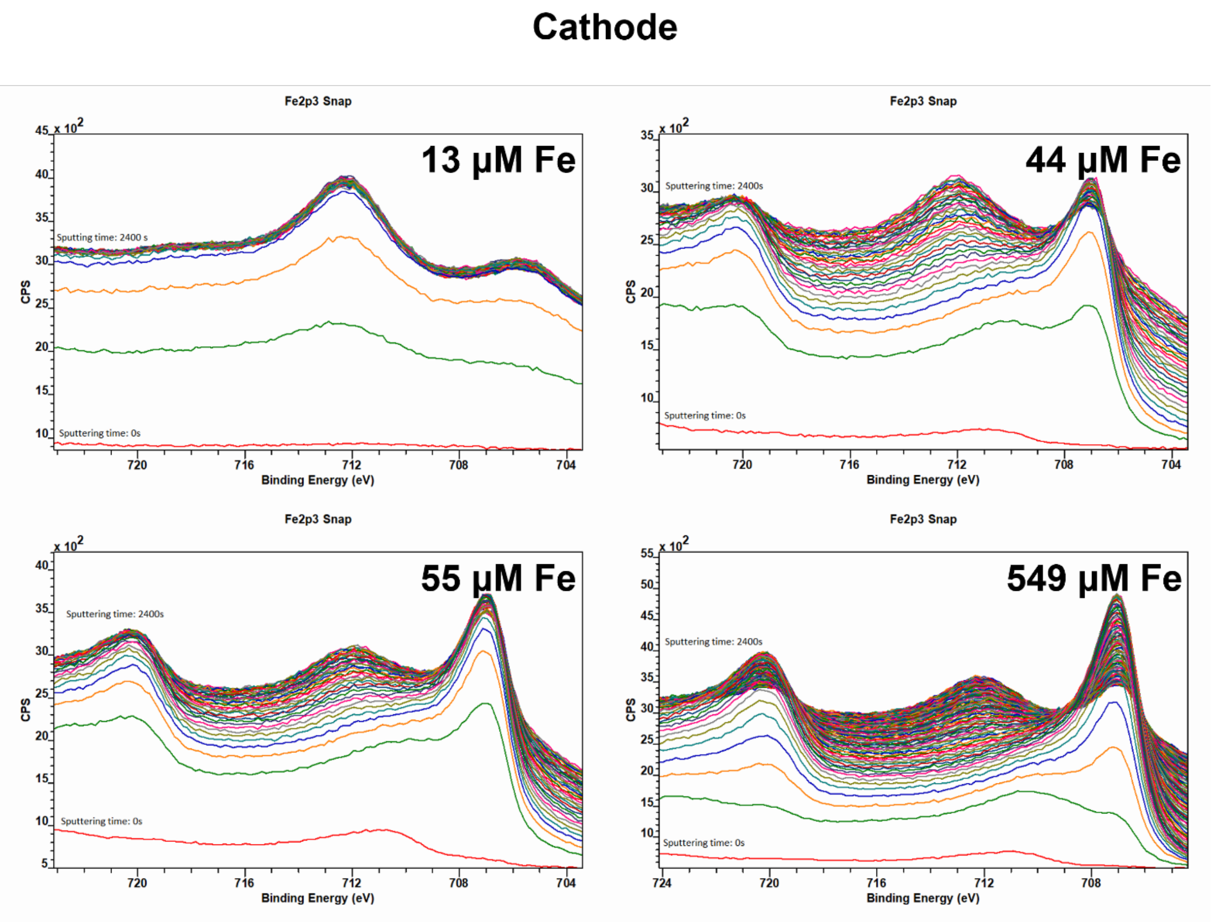

Figure S27. XPS spectra of the Fe-2p³ peaks of the cathode at 13, 44, 55, and 549 μM Fe concentration post electrolysis.

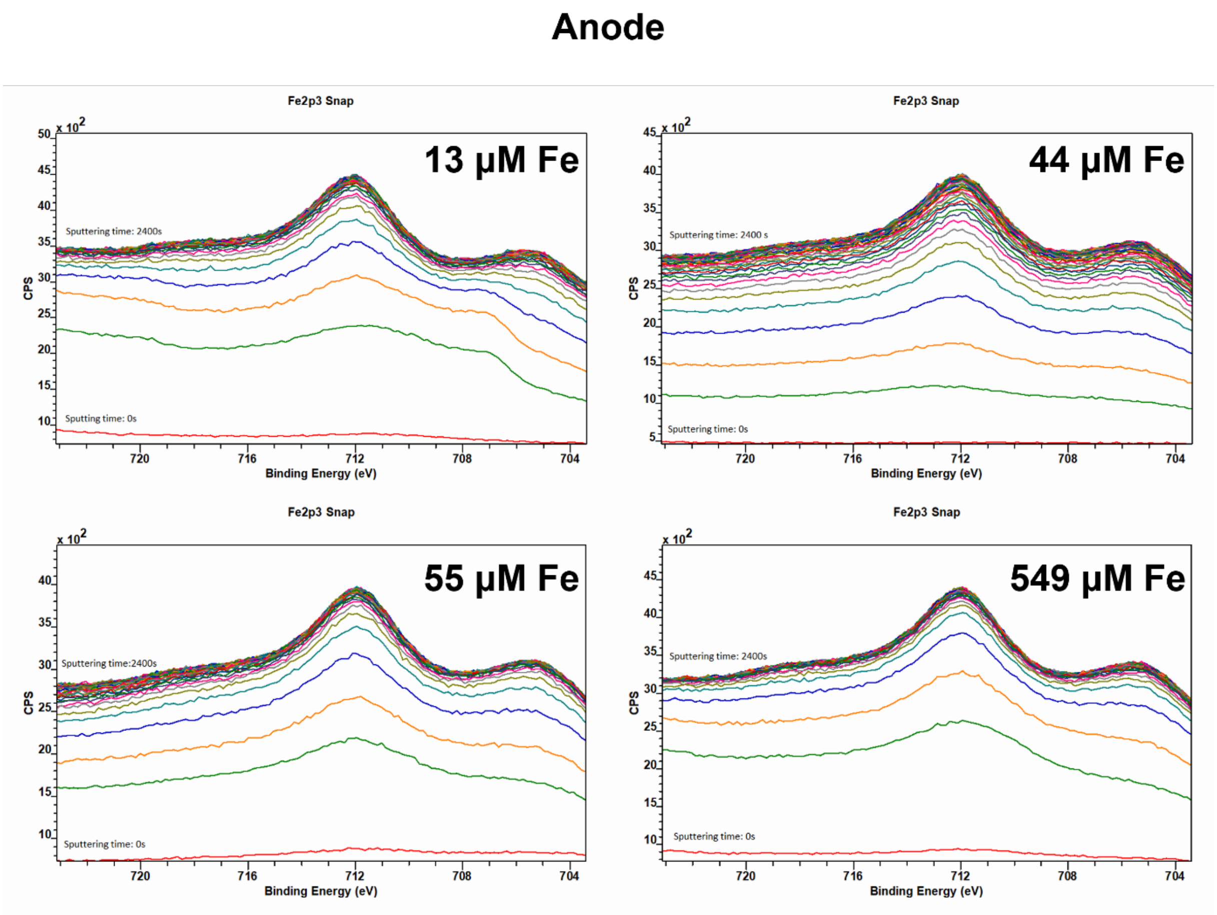

Figure 28. XPS spectra of the Fe-2p[3] peaks of the anode at 13, 44, 55, and 549 μM Fe concentration post electrolysis.

Cathode

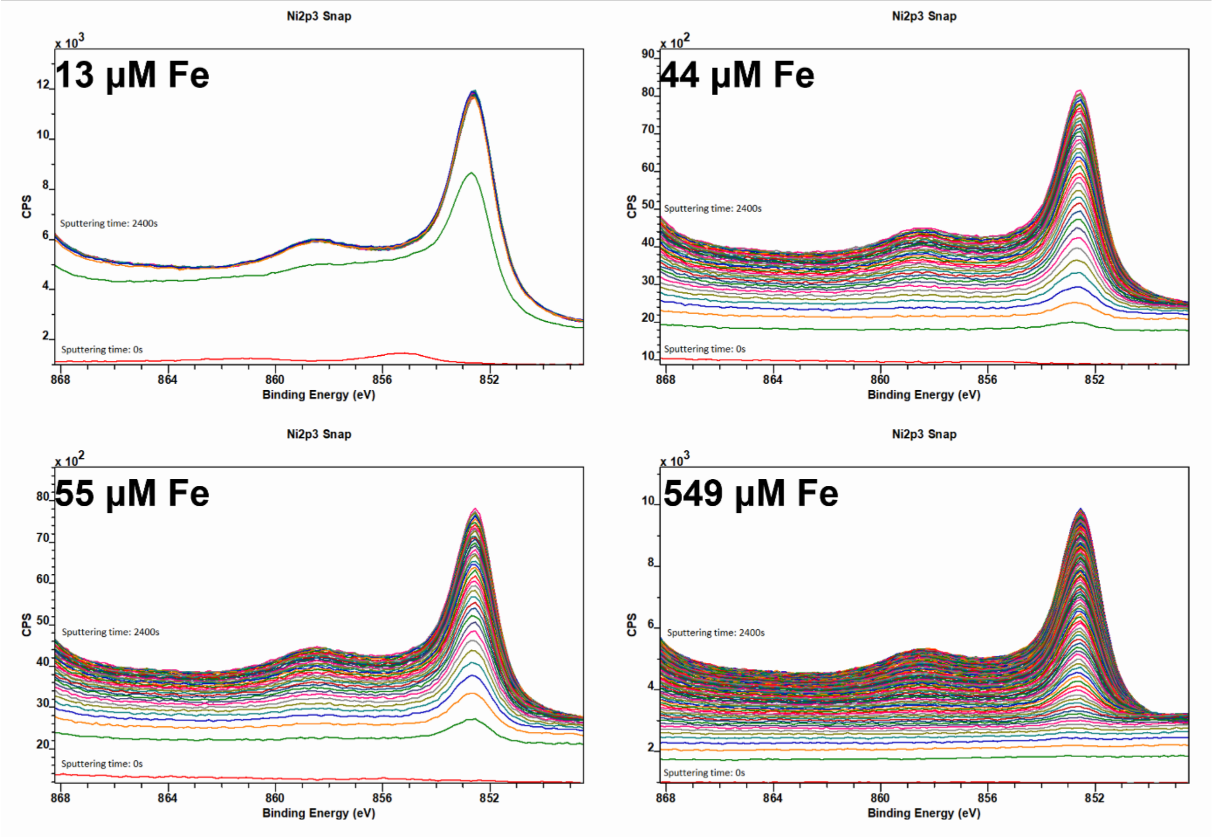

Figure S29. XPS spectra of the Ni-2p^3 peaks of the cathode at 13, 44, 55, and 549 µM Fe concentration post electrolysis.

Anode

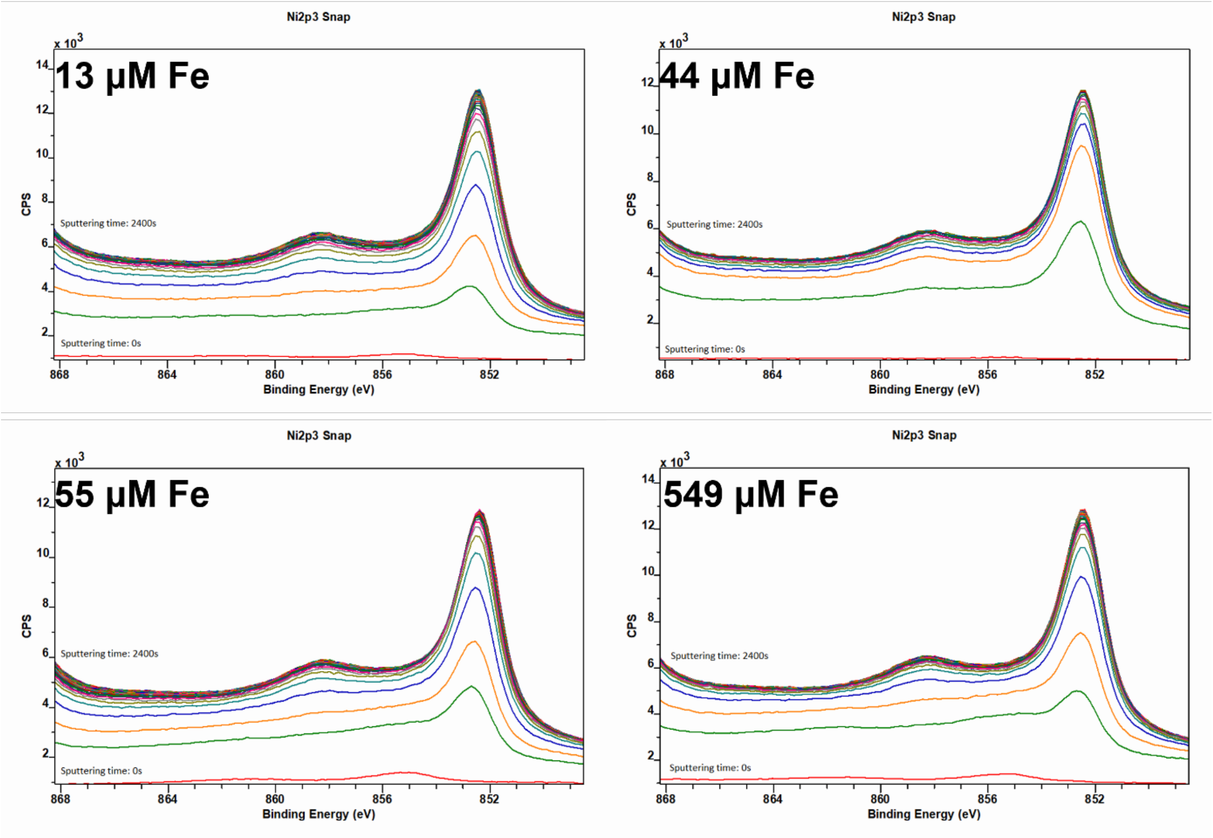

Figure S30. XPS spectra of the Ni-2p^3 peaks of the anode at 13, 44, 55, and 549 μM Fe concentration post electrolysis.
